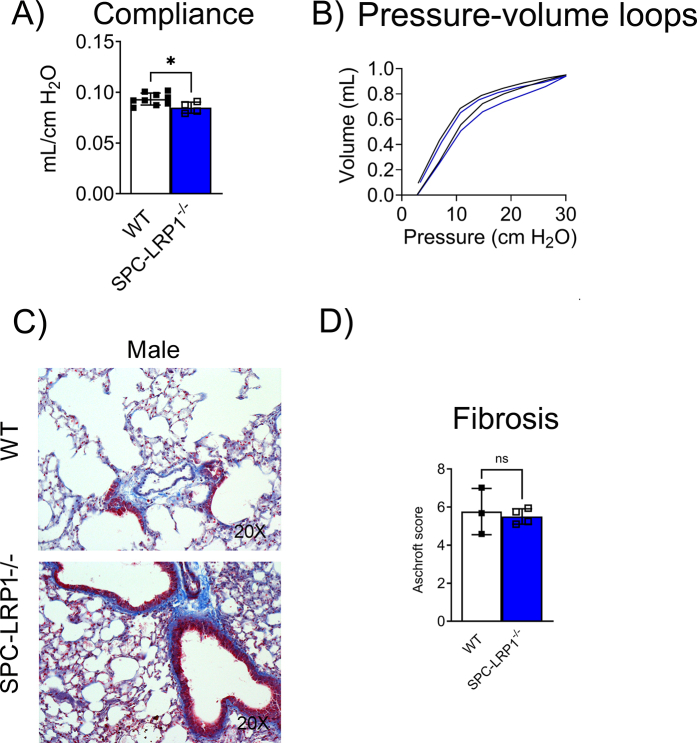# Alveolar type 2 cell LRP1 is needed for surfactant phospholipid metabolism and pulmonary function in mice

**DOI:** 10.1016/j.jlr.2026.101042

**Published:** 2026-04-16

**Authors:** Rafael Ward, Soumya S. Krishnan, Irina Stavrovskaya, Dativo Sanchez-Gonzalez, Christina W. Agudelo, Sangmi S. Park, Michelle Mai, Huchong Cai, Jennifer Martinez, Kimone Cox, Amrin Rahman, Christopher Kiang, Mattin Campos-Azpiroz, Patrick Geraghty, Elena Lopez-Rodriguez, Estela Area-Gomez, Igor Shmarakov, Ira J. Goldberg, Robert F. Foronjy, Itsaso Garcia-Arcos

**Affiliations:** 1 Departments of Medicine and Cell Biology, SUNY Downstate Health Sciences University, Brooklyn, NY, USA; 2 Institute of Functional Anatomy, Charité Universitätsmedizin, Berlin, Germany; 3 Departments of Pathology, Columbia University Medical Center, New York, NY, USA; 4 Department of Animal Sciences, Rutgers University, New Brunswick, NJ, USA; 5 Department of Medicine, NYU Langone School of Medicine, New York, NY, USA

**Keywords:** lipid, lipoprotein receptor, pulmonary surfactant

## Abstract

The low-density lipoprotein receptor-related protein 1 (LRP1) performs multiple functions with cell-specific regulation. Genetic variants in *LRP1* are associated with chronic obstructive pulmonary disease (COPD), but the underlying mechanisms are unknown. Alveolar type 2 cells (T2C) synthesize pulmonary surfactant lipids and are involved in the pathophysiology of COPD. To investigate LRP1 in T2C, we stably transfected a human T2C cell line with *LRP1*-shRNA (*LRP1* knockdown, *LRP1* KD) and generated tamoxifen-inducible T2C-specific LRP1 knockout mice (SPC-LRP1^−/−^). *LRP1* KD cells showed decreased surfactant phospholipid secretion and increased neutral lipid accumulation, despite lower expression of lipid metabolic genes. T2C and alveolar surfactant isolated from SPC-LRP1^−/−^ mice showed lower concentrations of phosphatidylcholine than those from *Lrp1*-floxed controls. At baseline, SPC-LRP1^−/−^ mice had decreased lung compliance and forced vital capacity. After a cigarette smoke exposure challenge, SPC-LRP1^−/−^ mice developed worse fibrotic remodeling than control mice. Both LRP1 KD cells and T2C isolated from SPC-LRP1^−/−^ mice exhibited increased gene expression of detoxification and inflammatory pathways associated with COPD. Finally, the query of public human data showed that T2C from patients with COPD have lower expression of *LRP1* and lipid metabolic genes. These data show that LRP1 is needed in T2C for surfactant lipid metabolism and pulmonary function, and suggest that reductions of LRP1 expression promote smoke-associated fibrosis.

The LDL receptor-related protein 1 (LRP1) is a transmembrane protein that was first described as the hepatic receptor mediating endocytosis of chylomicron remnants (1, 2). Besides this function, LRP1 participates in multiple other cellular processes that are regulated in tissue and cell-type-specific manners (3). The extracellular portion of LRP1 can bind more than 30 different ligands, including Apo-E containing lipoproteins, lipases, serine protease inhibitors and extracellular matrix (ECM) components (3). Illustrating the heterogeneity of its ligands and functions, LRP1 alternative names include alpha-2-macroglobulin receptor (A2MR), transforming growth factor-beta receptor type V (TGFBR5), and cluster of differentiation 91 (CD91). Recently, LRP1 was identified as the host receptor used for entry by the Rift Valley fever virus and other RNA viruses (4, 5, 6). The intracellular portion of LRP1 can bind to adaptor proteins that in turn regulate endocytic and signaling activities (7).

LRP1 has been mostly studied in the context of metabolic and cardiovascular disease. Genome-wide association studies (GWAS) showed a correlation between single-nucleotide polymorphisms (SNPs) in *LRP1* and coronary artery disease (8), aortic aneurysm (9), and blood lipid levels (10, 11). A new autosomal recessive LRP1-related syndrome comprising prematurity, severe respiratory distress syndrome, ascites and neurologic and cardiac developmental defects is associated with two SNP variants (12). The pathophysiology due to defects in LRP1 is, in most cases, undefined. Most LRP1 SNPs lead to lower expression of *LRP1* (9, 11, 12, 13, 14, 15, 16). Because total body *Lrp1* knockout is embryonic lethal in mice (17, 18, 19), tissue-specific knockout mice were used to show that LRP1 regulates lipid metabolism in liver (20, 21), adipose tissue (22) and macrophages (23, 24). Adipose-specific *Lrp1* knockout mice had delayed postprandial lipid clearance (22), human macrophages with *LRP1* deletion showed defective VLDL uptake and increased secretion of inflammatory cytokines (23, 24, 25), and LRP1-deficient hepatocytes had impaired HDL secretion (20).

LRP1 is associated with human lung disease. A SNP variant in *LRP1* was linked with decreased pulmonary function in smokers and patients with chronic obstructive pulmonary disease (COPD) (14, 26). This SNP occurs in an intronic regulatory sequence that binds to transcription factors and can regulate *LRP1* expression in some tissues (16). However, in the lung, the specific links with disease are unclear because LRP1 is present in multiple pulmonary cell types, and the regulation of its functions is not well understood. We recently showed increased LRP1 protein in airway epithelial cells from patients with COPD, and mice with airway epithelial cell-specific *Lrp1* knockout had worse fibrosis after smoke exposure, suggesting protective roles of LRP1 in the lung epithelium (27). LRP1 may be protective in alveolar macrophages by mediating efferocytosis (28). In alveolar macrophages isolated from COPD patients, cigarette smoke exposure decreased the expression of *LRP1* (29), and in pulmonary fibroblasts, LRP1 participates in tissue repair by controlling extracellular matrix remodeling (30, 31, 32). Despite its original identification as a lipid receptor, LRP1 has not been studied in the context of pulmonary lipid metabolism.

Alveolar type 2 epithelial cells (T2C) synthesize pulmonary surfactant, a lipoprotein complex that decreases alveolar surface tension during inspiration and prevents alveolar collapse during expiration. Surfactants are composed of ∼90% lipids, primarily phospholipid, and ∼10% specific surfactant proteins. Dipalmitoylphosphatidylcholine (PC 32:0) is the most abundant lipid in surfactants, followed by other phospholipids, cholesterol, and some sphingolipids (33). Surfactant deficiency causes infant respiratory distress syndrome in premature neonates (34, 35, 36). In adults, surfactant deficiency and altered lipid composition are associated with multiple lung diseases, including COPD and pulmonary fibrosis (33, 37, 38, 39, 40, 41, 42, 43). Mutations in proteins involved in surfactant homeostasis, such as surfactant protein C (SP-C) and intracellular phospholipid transporter ABCA3, cause surfactant deficiency in humans and pulmonary fibrosis in animal models (44). T2C-specific knockout of lipid metabolic genes such as cholesterol exporters *Abca1* and *Abcg1*, or the phospholipid synthesis rate-limiting enzyme CTP: phosphocholine cytidylyltransferase α (CCTα), all show impaired pulmonary function in animal models (45, 46, 47). LRP1 is expressed in T2C (48), but it has not been studied in the context of their lipid or surfactant metabolism.

We hypothesized that LRP1 is involved in lipid metabolism in T2C and is necessary for surfactant lipid homeostasis and pulmonary function. This function of LRP1 in T2C may explain the genetic association of LRP1 with COPD. We generated a stably transfected human T2C cell line with *LRP1* knockdown and cultured it at the air-liquid interface (ALI) to study surfactant secretion in vitro. We also generated tamoxifen-inducible T2C-specific *Lrp1* knockout mice (SPC-LRP1^−/−^) to study pulmonary function in vivo. Our data show that LRP1 is needed for maintaining alveolar surfactant phospholipid concentration in vitro and in vivo, that LRP1 deficiency in T2C causes overexpression of intracellular detoxification and inflammation pathways, and that loss of LRP1 in T2C exacerbates fibrotic lung tissue remodeling in secondhand smoke-exposed mice.

## Materials and methods

### Generation of stable transfected cell line and cultures at the air-liquid interface (ALI)

Human A549 cells (Millipore Sigma) were cultured in DMEM with 10% FBS and 1% penicillin/streptomycin at 37°C and 5% CO2. Semiconfluent T-25 flasks were transfected with 1.5 μg of lentiviral particles carrying scrambled shRNA or *LRP1* shRNA (Santa Cruz Biotechnology, TX), following the manufacturer’s protocol. Successfully transfected cells were selected by resistance to puromycin and subsequently propagated. Knockdown of LRP1 was confirmed by Western blotting (WB). For experiments, cells were first grown in submerged conditions and then transferred to commercially available collagen-precoated transwells (Corning Life Sciences) in 12-well plates at a density of 0.5·10^6^cells/well, or in 6-well plates at a density of 1·10^6^cells/well. After 24 h, the media from the bottom chamber was renewed, the media from the apical side was removed to expose the cells to air, and the cells were cultured for an additional 24 h in these conditions (air-liquid interphase: ALI). In all experiments, the media in the bottom well were renewed, and the apical side was washed with PBS the night before collection.

### Immunocytochemistry

Transwells with confluent cells were washed with PBS, fixed with 3.7% formaldehyde and permeabilized with 0.1% Triton X-100. They were blocked with 10% FBS or 3% BSA, and incubated with rabbit anti-LRP1 antibody (dilution 1:200) or anti-SP-C (dilution 1:100) overnight at 4°C. Transwells were then washed with PBS three times and incubated with Alexa fluor 488-conjugated anti-rabbit secondary antibody (dilution 1:200) for 1 h at room temperature, followed by nuclei staining with Hoechst (dilution 1:2000) for 5 min. For Oil Red O staining, a stock solution was first prepared by dissolving 300 mg of Oil Red O powder in 100 ml of 99% isopropanol. Transwells were fixed as above, washed with water, and incubated with 60% isopropanol for 5 min, followed by 5 min of incubation with Oil Red O working solution freshly prepared by mixing 3 parts of stock with 2 parts of water and filtering. Transwells were rinsed with tap water, counterstained with hematoxylin for 1 min and rinsed again with tap water. Transwell membranes were then carefully cut and mounted on coverslips with glycerin jelly. Images were acquired with a Nikon Eclipse 80i epifluorescence microscope or with an Axio Observer 7 LSL 800 confocal microscope (Carl Zeiss Microscopy). Antibodies used in this study are listed in Table 1.

**Table 1:** Antibodies used in this study

Target Protein	Antibody Details
b-Actin	Polyclonal, Cell Signaling Technology cat# 4967: β-Actin Antibody
CD32	Monoclonal, BD Biosciences, cat# 553143; BD Pharmingen™ Biotin Rat Anti-Mouse CD16/32
CD45	Monoclonal, BD Biosciences, cat# 553078; BD Pharmingen™ Biotin Rat Anti-Mouse CD45
GAPDH	Monoclonal, Cell Signaling Technology cat# 5174: GAPDH (D16H11) XP® Rabbit mAb
LRP1	Monoclonal, AbCam cat# ab92544: Anti-LRP1 antibody [EPR3724]
Surfactant protein A	Polyclonal, AbCam cat# ab103789: anti-surfactant protein A antibody
Surfactant protein B	Monoclonal, AbCam cat# ab3282: anti-surfactant protein B antibody
Pro-surfactant protein C	Polyclonal, AbCam cat# ab90716: anti prosurfactant protein C antibody
Surfactant protein D	Monoclonal, AbCam cat# ab168366: anti surfactant protein D antibody
Ep-CAM	Monoclonal, AbCam cat # ab313668: Anti-EpCAM antibody, Alexa Fluor 555 conjugated
Mouse IgG	Polyclonal, AbCam cat# ab6668: anti-mouse IgG antibody
Rabbit IgG	Polyclonal, Cell Signaling Technology cat# 7074: Anti-rabbit IgG, HRP-linked Antibody.
Rabbit IgG	Polyclonal, Vector Laboratories cat# BA-4000-1.5: Anti-Rat IgG Antibody (H + L), Biotinylated.
IgG2B	Monoclonal, R&D Systems, cat # MAB0061; Rat IgG2B Isotype Control
Rabbit IgG (F(ab’)2 fragment)	Polyclonal, Thermo Fisher Scientific cat # A11070: F(ab’)2 fragment of goat anti-rabbit IgG (H + L) secondary antibody; Alexa Fluor ^TM^ 488 conjugated.

### Generation of SPC-LRP1^−/−^ mice

All studies involving animals were approved by the IACUC at SUNY Downstate Health Sciences University. Tamoxifen-inducible T2C-specific *Lrp1* knockout mice (SPC-LRP1^−/−^) were generated by crossing commercially available *Lrp1*^*flox/flox*^ mice (Jackson Labs, ME) with *Sftpc-CreER*^*T2*^ mice generously donated by Dr Brigid Hogan (Duke University) to generate *Sftpc-CreER*^*T2*^*-Lrp1*^*flox/WT*^ (hemizygous) mice, which were crossed again with *Lrp1*^*flox/flox*^ mice to generate *Sftpc-CreER*^*T2*^*-Lrp1*^*flox/flox*^ mice (SPC-LRP1^−/−^). Activation of Cre recombinase and subsequent Lrp1 loss specifically in T2C was induced by 5 consecutive days of tamoxifen injection at 5–6 weeks of age, thus allowing full postnatal mouse lung development. SPC-LRP1^−/−^ mice and littermate controls *Lrp1*^*flox/flox*^ (WT) mice were born at the expected Mendelian ratio and did not show any visible phenotype during development. Both male and female mice were used, and unless otherwise indicated, mice were euthanized at 6 months of age. The findings in both male and female mice are presented, and sex-dimorphic effects are discussed in the text.

### Collection of mouse tissues and bronchoalveolar lavage (BAL)

Immediately after mouse euthanasia, BAL fluid was collected by instilling and retrieving 1 ml of PBS through a tracheal catheter. Blood was collected from the heart, and cold PBS was perfused through the ventricles until the livers and lungs were clear. Tissues were then harvested, flash-frozen and stored at −80°C for later analysis. BAL fluid and BAL cells were separated by centrifugation at 300xg for 10 min, and BAL cells were counted in a Neubauer chamber. In some experiments, lungs were pressure-perfused with 10% formalin following the guidelines by the American Thoracic Society for sectioning and histology (49).

### Surface tension calculation

Surface tension in BAL was assessed by capillary rise (50). Briefly, an aliquot of 100 μl of BAL fluid was warmed to 37°C, and a capillary was introduced into the tube to allow for the BAL fluid to rise into the tube by capillarity. The height of BAL fluid in the capillary was then measured, and surface tension calculated using Jurin’s law: h = 2γcosθ/ρɡr_0_ where h is the measured height, γ is the surface tension, θ is the angle of contact of the BAL with the wall of the capillary, ρ is the calculated density of the BAL fluid, ɡ is the gravitational force 9.8 m/s^2^, and r_0_ is the radius of the capillary 0.055 mm. Data were then normalized to WT mice.

### Isolation of primary T2C

Primary T2C were isolated as in Corti *et al*. (51). Briefly, mice were anesthetized, perfused as above, and 1 ml of dispase in DMEM was instilled through the tracheal catheter, followed by 0.5 ml of 1% low melting point agarose at 40°C. Lungs were then covered with ice for 2 min to solidify the agarose. The lungs were then excised and incubated in dispase for 45 min with gentle rocking, after which they were transferred to 0.01% DNase I in DMEM and gently shaken manually. The resulting cell suspension was filtered through 100 and 40 μm cell strainers and pelleted by centrifugation at 130 x g for 8 min at 4°C. Pelleted cells were treated with red blood cell lysis buffer, incubated with biotin-conjugated anti-CD45 and anti-CD16/32 antibodies and then with streptavidin-coated magnetic beads for negative selection of T2C by magnetic column chromatography. The flow through containing T2C was collected, cells were pelleted, and frozen until further use.

### RNA sequencing and pathway analysis

Primary T2C were isolated as above, and dry pellets (N = 3–5/group) were submitted for mRNA sequencing (Genewiz, Azenta Biosciences) (52). Differential gene expression and pathway analysis were conducted using Express Analyst Pro and following the recommended protocol (53). Briefly, raw counts were uploaded to the software, features filtered with a cutoff of 15 for variance and 4 for abundance and normalized using the Relative Log Expression method. Differential expression analysis was performed with the Limma method. All features were considered for the Volcano plot and downstream pathway analysis. Gene set enrichment analysis (54) was conducted using the KEGG library as a reference pathway database and the gene set library as a background universe. Significantly different pathways (*P*< 0.05) were visualized in a ridgeline plot.

### Pulmonary function assessment

Pulmonary function testing was performed by forced oscillatory maneuvers using a Flexivent system (SciReq) as in (27). Briefly, mice were anesthetized and an 18-gauge blunt catheter was inserted through the trachea. Mechanical ventilation was initiated, and mice were paralyzed with an intramuscular injection of pancuronium bromide. The animal was ventilated at a respiratory rate of 150 breaths/min and tidal volume of 10 ml/kg against a positive end-expiratory pressure of 3 cm H_2_O. The manufacturer’s protocol was followed for single-frequency and low-frequency force oscillatory technique, deep inflation, pressure-volume loops, and force expirations. These measurements are automated and determined multiple times per animal over a 3-min period.

### Lipidomic analysis

Lipid extracts were prepared by chloroform-methanol extraction, spiked with appropriate internal standards, and analyzed using a 6490 triple quadrupole lipid chromatography/mass spectrometry (LC/MS) system (Agilent Technologies, Santa Clara, CA) as described (55). Glycerophospholipids and sphingolipids were separated with normal-phase HPLC using Agilent Zorbax Rx-Sil columns (inner diameter 2.1 x 100 mm) under the following conditions: mobile phase A (chloroform:methanol:1 M ammonium hydroxide, 89.9:10:0.1, v/v/v) and mobile phase B (chloroform:methanol:water:ammonium hydroxide, 55:39.9:5:0.1, v/v/v); 95% A for 2 min, linear gradient to 30% A over 18 min and held for 3 min, and linear gradient to 95% A over 2 min and held for 6 min. Quantification of lipid species was accomplished using multiple reaction monitoring transitions developed in earlier studies in conjunction with internal standards references (Avanti Polar Lipids) as in (37). Results were then normalized by cell number or protein amount for intracellular lipids, or by epithelial lining fluid (ELF) for BAL lipids as in (37).

### RNA extraction, cDNA synthesis, and qPCR

The protocol as in (56) was followed. RNA was extracted from each sample using commercially available reagents (Zymo Research, CA), cDNA was synthesized (ThermoFisherJ), and qPCR was performed using SYBR Green Master Mix (Selleck Chemicals, TX). Primers were designed using OligoPerfect Designer Tool (ThermoFisher), and the sequences are listed in Table 2.

**Table 2:** Sequence of primers used in the study

Species	Gene Name	Forward Sequence	Reverse Sequence
Human	ABCA1	CATGATCTGGAAGGCATGTG	ATGTCTTGGGCTGTCCAATC
	ABCA3	GTCGTGCAGGAGAAGGAAAG	GTTGGCTTTGCTGAAGAAGG
	ABCG1	ACGCAGTTCTGCATCCTCTT	CGGAGTTGCTCAAGACCTTC
	ACACA	CAAGGAGGTGAAGGACAAGC	ATTGGACATGCTCTCCATCC
	ACOX1	CGTGAAACCGCTGAGTAACA	TTCAGACTGGTGCCTCACAG
	Actin	GAAGGTGACAGCAGTCGGTT	AGGGACTTCCTGTAACAATGC
	CCTa	GCAAACTCCCACAATGAGGT	GCAACCAGCTCCTTTTTCTG
	CK	TGTATGGAGCGATTTTGCAG	TGGGGAAAGATGCCATAGAG
	CPT1b	AGGCCTCAATGACCAGAATG	CACCTCAGCAAGGAAAGGAG
	DGAT1	CATCCTGAACTGGTGTGTGG	AAGACATTGGCCGCAATAAC
	DGAT2	AAAGAATGGGAGTGGCAATG	CAGGTGTCGGAGGAGAAGAG
	FASN	GTTTGATGCCTCTTCTTCG	CGGAGTGAATCTGGGTTGAT
	HMGcoA Reductase	ACGGTATGCCCTGGTAGTTG	ACTGGGCATGGATCTTTTTG
	HMGCoA synthase	ACGGTATGCCCTGGTAGTTG	CACTGGGCATGGATCTTTTT
	PGC1b	GGAGACTGAACCCTGAGCTG	GTAGGAGCTGCTGGTCTTGG
	PLA2	TTCTGATCCCCAATGCTTC	AGTTAAGGGCCAGACCCAGT
	PPARa	ACGATTCGACTCAAGCTGGT	GTTGTGTGACATCCCGACAG
	SCD1	CCCAGCTGTCAAAGAGAAGG	GGGGGCTAATGTTCTTGTCA
	SREBP1	AACCATCTTGGCAACAGTCC	AATGTAGTCGATGGCCTTGC
	SREBP2	GACATCATCTGTCGGTGGTG	GGGCTCTCTGTCACTTCCAG
Mouse	*Ccl2*	AGCACCAGCCAACTCTCACT	CGTTAACTGCATCTGGCTGA
	*Cd68*	CCAATCAGGGTGGAAGAAA	CTCGGGCTCTGATGTAGGTC
	*Col1a1*	CACCCCAATCTGGTTCCCTC	CATAAGCCAAGTGGGCAGGA
	*Col1a2*	CCCGTTGGCAAAGATGGTAG	ACCTTGGCTACCCTGAGAAC
	*Col3a1*	ACTGGTGAACGTGGCTCTAA	AACCTGGAGGACCTGGATTG
	*Il-6*	CCAGGCCTACACTCCCTACT	CAGAGTGTGGCAGTGGGAAT
	*KC*	GTTCCAGCACTCCAGACTCC	CCTCGCGACCATTCTTGAGT
	*TNFa*	AGCCCCCAGTCTGTATCCT	CTCCCTTTGCAGAACTCAG

### Fatty acid oxidation activity

Confluent transwells of control and LRP1 KD cultures were incubated with serum-free media for 1h. Media were then renewed and supplemented with ^3^H-labelled palmitic acid (0.1ul/ml) and 1.5 mM unlabeled palmitic acid complexed with fatty-acid free-BSA (2:1 ratio), and incubated with the cells for 30 min. A batch of transwells was harvested for reference at time 0, and in another batch, the media were refreshed, transwells washed and cells collected 1h later. Intracellular lipids were extracted, and ^3^H was measured in the organic phase and normalized to cell number and ^3^H at time 0.

### Free fatty acid uptake

Commercially available reagents were used, and the manufacturers’ protocol was followed (AbCam). Briefly, confluent transwells of control and LRP1 KD cells were incubated with serum-free media for 1h. The media were then replaced and supplemented with Bodipy-labelled C12 fatty acid. Cells were collected at time points 0, 1, 15 and 20 min for measurements of fluorescence and protein concentration.

### Fatty acid synthase activity

The enzymatic activity of FASN was assessed using a commercially available kit (Abbkine, GA). Briefly, 2 million control and LRP1 KD cells per replicate were collected and processed according to the manufacturer’s protocol. The absorbance value at 340 nm was measured at 37°C and the resulting activity values were normalized to protein concentration to calculate enzymatic specific activity.

### Western blot

Cell pellets or tissues were homogenized in 4 volumes of RIPA buffer containing protease and phosphatase inhibitors, and equal protein amounts were loaded into polyacrylamide gels. For BAL, equal volumes of lavage were loaded in the gel. SDS-PAGE was conducted, and proteins were transferred to nitrocellulose membranes. Membranes were then blocked with 5% BSA in TBST for 1 h, washed three times with TBST, incubated with primary antibody at a dilution 1:1000 in 2% TBST overnight at 4°C. Membranes were then washed three times with TBST and incubated with horseradish peroxidase-linked secondary antibody at a dilution 1:4000 in 1% TBST for 1 h, and washed three times. Membranes were developed using chemiluminescent substrate (ThermoFisher). The antibodies used in this study are listed in Table 1.

### Histology

Fixed mouse lungs were processed for paraffin embedding, followed by sectioning and staining with H&E or Trichrome, or for OCT-embedding followed by cryosectioning (Horus Scientific, MA). Cryosections were used for Oil Red O staining in combination with immunohistochemistry for CD45 using commercially available reagents (Vector Laboratories, CA). Briefly, sections were blocked with normal rabbit serum for 2 h and then incubated with 10 mg/ml of anti-mouse CD45 for 2 h. For negative controls, the primary antibody was replaced with rat IgG2B isotype control (R&D Systems) at the same concentration as the primary antibody. Endogenous peroxidase was blocked with 3% H_2_O_2_ in methanol for 3 min. Sections were then incubated with biotin-labeled rabbit anti-rat IgG serum for 30 min, followed by avidin-biotin-alkaline phosphatase complex treatment for 30 min, washed in 0.1 M PBS, and developed in DAB chromogen solution (Vectastain ABC kit). Oil red O stain was then conducted according to the manufacturer’s protocol (Sigma Aldrich, USA). Slides were sequentially incubated in 2-propanol 60% for 20 s, Oil red O staining solution for 10 min, 2-propanol 60% for 30 s, and distilled water for 20 s. Finally, slides were counterstained with hematoxylin (Abcam, USA) for 3 min, rinsed with tap water, allowed to dry and mounted in glycerin jelly. Sections were imaged by light microscopy at 20X. For each mouse lung section, 10 non-contiguous fields were captured and used for counting cells positive for CD45, Oil Red O, or both.

For immufluorescence staining, slides were deparaffinized by incubation in Histoclear, followed by sequential dehydration in ethanol 100%–80% and water rinse. Antigen retrieval was performed in tri-sodium citrate buffer for 10 min at 95°C in a water bath. After cooling to room temperature and washing with Tween 0.1% (w/v)-supplemented phosphate-buffered saline with (PBST), tissues were permeabilized with 0.5% Triton X-100 for 10 min. Slides were washed again with PBST, incubated with 3% H_2_O_2_ for 5 min for blocking endogenous peroxidase activity, and then incubated in 3% BSA for 1h at room temperature, followed by overnight incubation with rabbit anti-LRP1 antibody (dilution 1:100) at 4°C. Slides were then washed with PBST, incubated with Alexa 488-conjugated anti-rabbit IgG (dilution 1:200) for 1h at room temperature, and thoroughly washed again with PBST. Slides were then incubated with Alexa fluor 555-conjugated anti-EpCam antibody for 2h at room temperature and then washed with PBST. Nuclei were then stained with Hoechst in PBS for 10 min. Slides were washed, allowed to dry, and mounted in Prolong Gold antifading agent.

### Fluorescence confocal microscopy

Images were acquired using a Zeiss LSM800 Confocal Microscope with 20X and 63X objectives and an identical laser gain setting for all captures. Images were captured as a single snapshot and analyzed using Fiji (ImageJ) software following the software guides (57). For quantification of LRP1 signal, images were first converted to 8 bit and background subtraction with a rolling ball radius of 5 pixels was applied uniformly to all 488 nm channel images. AT2 cells were identified as alveolar, EpCAM-positive, and having a rounded or cuboidal morphology. Cell boundaries were confirmed using corresponding differential interference contrast images and regions of interest (ROI) were manually defined to correspond to T2C cell boundaries. Automated thresholding was performed using Otsu’s method, with visual verification, followed by automated particle analysis. LRP1 fluorescence intensity was quantified as integrated density within the defined ROI.

### Assessment of emphysema by calculation of chord length

H&E−stained lung sections were imaged at 20X by light microscopy. 10–15 images were collected per mouse, excluding areas with large blood vessels to avoid overestimation of chord length. Automated quantification was used employing Fiji image processor (57) with the MLI plugin, following the protocol explained by Crowley *et al*. (58). Briefly, horizontal and vertical test lines were created for each image, followed by transformation to an 8-bit image and Huang thresholding. Test lines were overlaid on the lung image, and the chords to be determined were separated by color thresholding. Measurement parameters were selected for chord lengths to be measured using horizontal and vertical test lines separately. Obtained chord lengths were plotted and further analyzed with GraphPad Prism.

### Ashcroft fibrosis scoring

Masson’s trichrome-stained paraffin sections of lungs were thoroughly analyzed following the method as in (59). Briefly, 30–50 images corresponding to non-contiguous fields were acquired per slide using a light microscope equipped with a 10X objective. Each image was separately evaluated and given a score between 0 and 8. The entire area of the image was used for the determination of the score, and a score of 0 was chosen if normal tissue prevailed in the field. The mean score of all the fields was considered the fibrosis score for each mouse. The representative images shown were acquired with a 20X objective for illustrative purposes.

### Transmission electron microscopy

Immediately after euthanasia, lungs were inflated with 0.5 ml of air and perfused with 4% formaldehyde in PBS through the heart. Lungs were then excised, submerged in fixative containing 2.5% glutaraldehyde in PBS for 24h, and dissected into 2 mm^3^ cubes. Samples were then washed in potassium buffer 3 times for 15 min each, post-fixed with 1% OsO4 for 1.5–2 h, dehydrated in ethanol solutions, and embedded in Epon 812 as in (60). Ultrathin sections of 70 nm were cut using an ultramicrotome (Leica) equipped with an ultra-45° diamond knife (Diatome), collected on pioloform-coated copper grids, and subsequently stained with lead citrate according to Reynolds (61). Visualization was performed by using a Zeiss Leo 906 transmission electron microscope at 80 kV acceleration voltage and a Zeiss Leo 912 Ω transmission electron microscope at 120 kV acceleration voltage, both equipped with a slow scan 2K CCD camera (TRS).

### Stereological analysis

Systematic uniform random area sampling (SURS) was used to generate TEM micrographs from 3-5 sections per lung, and at least 50–60 fields of view were analyzed to obtain 100-200 counting events per lung. For superimposing a point and line test system, a STEPanizer stereology tool (62) was used. The measured parameters and calculations are detailed in Table 3.

**Table 3:** Stereological parameters measured and equations used

Parameter	Test System Used	Equation
Volume fraction of T2C in parenchyma (V_V_(T2C | par))	Point counting	V_V_(T2C | par) = P(T2C)/P(par)
Volume fraction of collagen in parenchyma (V_V_(coll | par))	Point counting	V_V_(coll | par) = P(coll)/P(par)
Volume fraction of lamellar bodies (LB) in T2C (V_V_(LB | T2C))	Point counting	V_V_(LB | T2C)=P(LB)/P(T2C)
Surface area density of LB Sv (LB)	Intersection counting	Sv (LB) = (2∗0.36∗P(LB))/(2∗I(LB))

### Statistics

Unbiased analysis for the intracellular lipidomic data was conducted using the software Metaboanalyst Pro and following the protocols as in (63). Briefly, for each lipid class, lipid concentrations were uploaded for principal component analysis and its visualization on scores and loading plots. Metaboanalyst was also used for generating a Vulcano plot of the differentially quantified lipid classes. All other statistical analyses were performed with GraphPad Prism. Mean values ± standard deviation (SD) are reported. Means were compared by two-tailed *t* test or a 2-way ANOVA. Data that did not follow normal distribution were analyzed with non-parametric tests and are presented as violin plots. Significances are reported as ∗*P*< 0.05, ∗∗*P*< 0.005, ∗∗∗*P*< 0.0005, and ∗∗∗∗*P*< 0.0001.

### Study approval

Experiments involving animals were approved by the IACUC at SUNY Downstate Health Sciences University.

## Results

### LRP1 knockdown impairs surfactant phospholipid metabolism in vitro

**Fig. 1:**
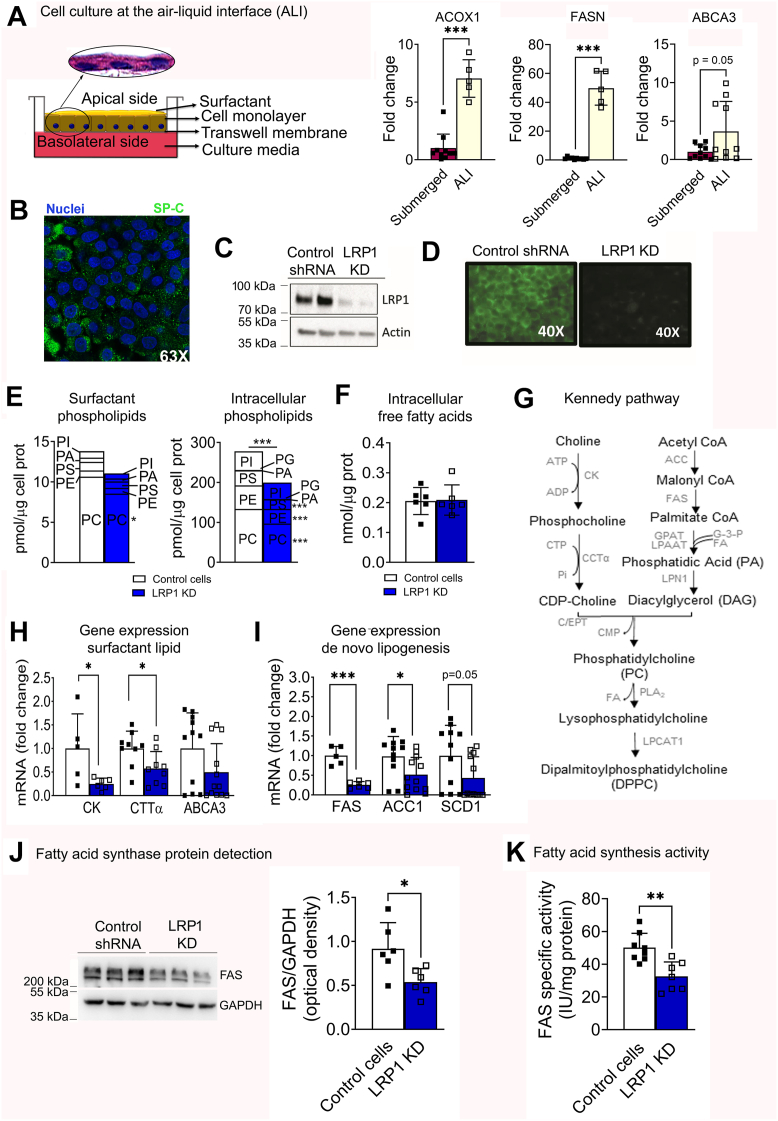
LRP1 knockdown (LRP1 KD) impaired surfactant lipid metabolism. Human A549 cells were cultured at the ALI and transfected with lentiviral vectors for knocking down LRP1 expression. Gene and protein expression and surfactant lipids were analyzed. A: Schematic of the cell culture model on collagen-coated transwells and maintained at the air liquid interface (ALI), and comparison of gene expression for selected lipid and surfactant-relevant proteins in cells cultured in submerged conditions vs. ALI. B: Confocal microscopy image of fluorescent staining for surfactant protein C (SP-C, green) and nuclei (Hoechst, blue) of cells cultured at the ALI. C: Confirmation of LRP1 knockdown in cells after stable transfection with LRP1 shRNA by Western blot and D: by immunofluorescence. Cells transfected with scrambled shRNA were used as controls. E: Phospholipid (PL) classes collected in the apical wash (surfactant) and cellular lysate of control and LRP1 KD cells cultured at the ALI. F: Intracellular concentration of free fatty acids (FFA). G: Schematic representation of the Kennedy pathway for de novo synthesis of dipalmitoylphosphatidylcholine (DPPC). H: Gene expression of enzymes involved in surfactant PL synthesis. I: Gene expression of enzymes involved in de novo lipogenesis. J: Representative Western blot for protein detection of fatty acid synthase (FAS) and optical density quantification of two independent experiments. K: Fatty acid synthase specific activity in lysates of control and LRP1 KD cells. Mean ± SD is shown. N ≥ 5 for all groups. Significance: ∗*P*< 0.05, ∗∗*P*< 0.01, ∗∗∗*P*< 0.001. ABCA3, ATP-Binding Cassette Subfamily A Member 3; ACACA, Acetyl-CoA carboxylase 1; ACOX1, acyl-Co oxidase 1; ALI, air liquid interface; CCTα, CTP:phosphocholine cytidylyltransferase alpha; CK, choline kinase; DAG, diacylglycerol; FASN, fatty acid synthase; FFA, free fatty acids; LPC, lysophosphatidylcholine; PA, phosphatidic acid; PC, phosphatidylcholine; PLA_2_, Phospholipase A_2_; SCD 1, stearoyl-CoA desaturase 1.

**Supplemental Figure S1:**
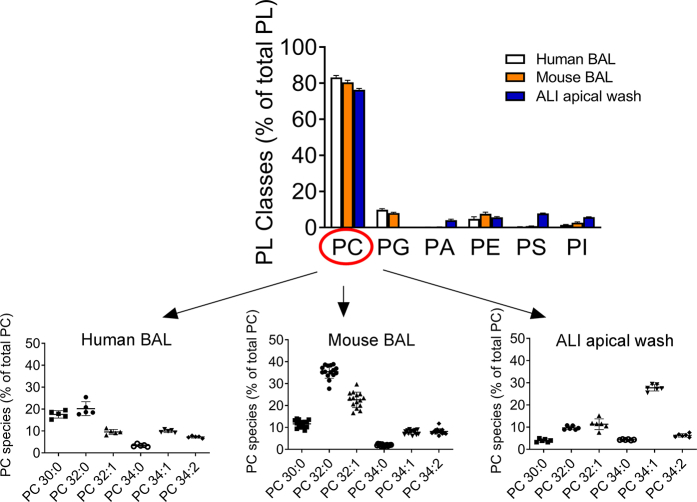


Studying surfactant metabolism in vitro is technically challenging because cultured T2C dedifferentiate over time in culture. However, human T2C-derived A549 cells form multivesicular bodies and synthesize surfactant phospholipid and proteins when cultured in transwells (64, 65, 66, 67, 68). Providing collagen substrates in regular cultures or culturing at the air-liquid interface (ALI) also promotes differentiation of T2C (69). To optimize the in vitro conditions for the study of surfactant, we used collagen-coated transwells (Corning Costar) and maintained the cultures at the ALI (Fig. 1A). Transferring the cultures from submerged conditions on plastic surface to ALI on collagen-coated transwells drastically increased mRNA expression of lipid metabolic genes, such as acyl-CoA oxidase (*ACOX1*; 7-fold; *P*< 0.001), fatty acid synthase (*FASN*; ∼50fold; *P*< 0.001), and of *ABCA3* (∼4-fold; *P*= 0.052), the T2C-specific lipid transporter into lamellar bodies specialized in surfactant storage (Fig. 1A). Surfactant protein C (*SP-C*) expression in these cultures was confirmed by immunofluorescence (Fig. 1B). Cells at the ALI visibly secreted a fluid coating to the apical side, resembling surfactant lipid. Indeed, more than 75% of the PL in the apical secretion was composed of PC, while other PLs such as phosphatidic acid (PA), phosphatidylethanolamine (PE), phosphatidylserine (PS) and phosphatidylinositol (PI) were detected in minor amounts (Supplemental Fig. S1). Amongst the PC species, the majority (>70%) was comprised of 16–18 carbon-length saturated or monounsaturated acyl chains (Supplemental Fig. S1). The PL composition was comparable to that of native surfactant from mice and humans, and we used this cell culture model for in vitro studies of LRP1 in T2C surfactant PL metabolism. To further assess the contribution of LRP1 to surfactant metabolism, an *LRP1* knockdown cell line (*LRP1* KD) was generated by stable transfection with lentiviral vectors containing an *LRP1*-specific shRNA sequence, and a control cell line was generated with lentiviral vectors containing scrambled shRNA (control cells). *LRP1* KD cells showed ∼90% LRP1 protein deletion by WB (Fig. 1C) and immunofluorescent staining (Fig. 1D).

*LRP1* KD decreased total surfactant PL recovered in the apical wash by ∼20% (Fig. 1E). The most significantly decreased PL was PC, declining from 10.6 ± 3.6 pmol/μg of cell protein in control cells to 8.5 ± 3.5 pmol/μg of cell protein in *LRP1* KD cells (*P*< 0.05; mean ± SD). The concentration of all other classes of PL decreased without reaching statistical significance (Supplemental Table S1). Extracellular lysophospholipids did not show proportionally increased concentrations (Supplemental Table S1), thus excluding PL degradation by extracellular phospholipases as an explanation for the decrease. Instead, intracellular PL was also decreased in *LRP1* KD cells, by ∼30% (*P*< 0.005; Fig. 1E). PC decreased from 133.5 ± 21 pmol/μg in control cells to 97.1 ± 9 pmol/μg cell protein in *LPR1* KD cells (*P*< 0.001; mean ± SD), and this was mainly attributable to the most abundant PC species (PC 30:0, 32:0 and 34:1), although there was a trend for all PC species to show lower values in *LRP1* KD cells. Some species of PLs also showed significant decreases, but the intracellular concentration of lysophosphatidylcholine and intracellular free fatty acids (FFAs) was unchanged (Fig. 1F and Supplemental Table S2).

Lower amounts of intracellular surfactant PC suggested its decreased synthesis by the Kennedy pathway (Fig. 1G). In *LRP1* KD cells, mRNA expression of *CCTa*, the rate-limiting enzyme for PC synthesis, was decreased by ∼50% (*P*< 0.05, Fig. 1H). Similar decreases were observed for choline kinase (*CK*), which catalyzes the committed step of choline to PC synthesis (*P*< 0.05), and for *ABCA3*, the lamellar body-specific lipid transporter (*P*= 0.052, Fig. 1 H). The mRNA expression of enzymes involved in de novo lipogenesis was also downregulated (Fig. 1I). Specifically, fatty acid synthase (*FASN*) decreased by 75% (*P*< 0.001), acyl-CoA acyltransferase (*ACC*) by 50% (*P*< 0.05), and stearoyl-CoA desaturase 1 (*SCD1*) by 60% (*P*< 0.05) in LRP1 KD cells (Fig. 1I). Consistently, protein levels for FAS were ∼0.5-fold lower, and FAS specific activity was ∼40% lower in LRP1 KD than in control cells (Fig. 1J–K). Therefore, the decrease in extracellular and intracellular surfactant lipid in T2C lacking LRP1 was associated with decreased expression and activity of the enzymes needed for PC synthesis. These data show that loss of LRP1 decreased extracellular and intracellular PL levels, in association with decreased de novo lipogenesis.

### LRP1 KD increased intracellular neutral lipid accumulation in vitro

**Fig. 2:**
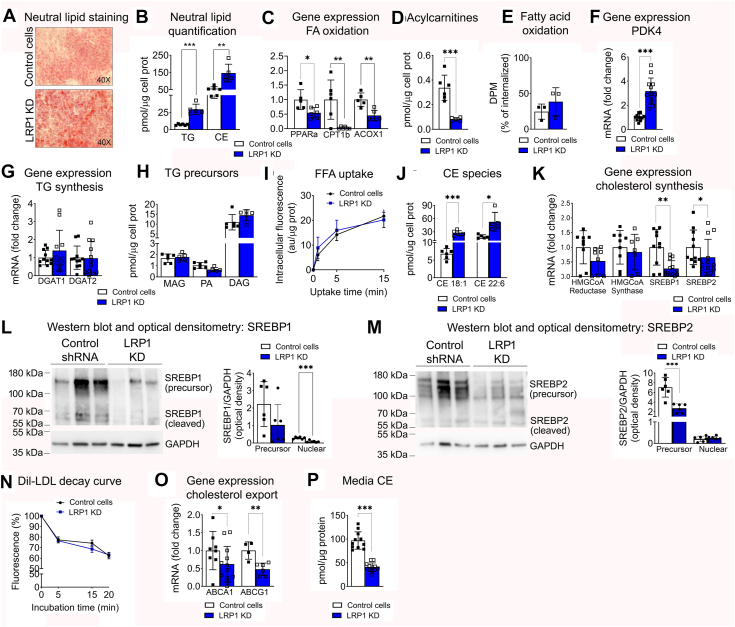
LRP1 knockdown increased intracellular neutral lipid storage. Control and LRP1 KD cells were cultured at the ALI and neutral lipid metabolism was analyzed. A: Representative images of Oil Red O staining for neutral lipids. B: Intracellular concentration of TG and CE. C: Gene expression of fatty acid oxidation-involved proteins PPARa, CPT1b and ACOX1. D: Concentration of intracellular acylcarnitines. E: Determination of fatty acid oxidation in cell lysates F: mRNA expression of PDK4. G: mRNA expression of TG synthesizing enzymes DGAT1 and DGTA2. H: Intracellular concentration of the TG precursors MAG, PA and DAG. I) Kinetics of cellular uptake of fluorescently labelled free fatty acid (FFA) by cultures at the ALI. J: Intracellular concentration of the most abundant cholesteryl esters CE 18:1 and CE 22:6. K) mRNA expression of the enzymes involved in regulating cholesterol synthesis HMG-CoA reductase, HMG-CoA synthase, SREBP1 and SREBP2. L-M: Representative western blots for SREBP1 and SREBP2 in their precursor and cleaved (active, nuclear) forms, with quantification of western blots from two independent experiments. N: Decay curve showing clearance from the media for fluorescently labelled Dil-LDL in control and LRP1 KD cultures. O: Gene expression of cholesterol exporters ABCA1 and ABCG1. P: Concentration of cholesterol ester in media after 16h of culture with control and LRP1 KD cells. Mean ± SD is shown. Significance: ∗*P*< 0.05, ∗∗*P*< 0.01, ∗∗∗*P*< 0.001. ABCA1, ATP Binding Cassette Subfamily A Member 1; ABCG1, ATP Binding Cassette Subfamily G Member 1; ACOX1, Acyl-CoA oxidase1; CE, cholesterol ester; CPT1b, carnitine palmitoyltransferase 1 beta; DAG, diacylglycerol; DGAT1, diacylglycerol O-acyltransferase 1; DGAT2, diacylglycerol O-acyltransferase 2; FFA, free fatty acid; GAPDH, Glyceraldehyde 3-phosphate dehydrogenase; HMG-CoA, hydroxymethylglutaryl-CoA; LDL, Low Density Lipoprotein; MAG, monoacylglycerol; PA, phosphatidic acid, PDK4, Pyruvate dehydrogenase kinase 4; PPARa, peroxisome proliferator-activated receptor alpha; SREBP, sterol regulatory element-binding transcription factor; TG, triacylglycerol.

Light microscopy indicated larger and more numerous intracellular lipid droplets in *LRP1* KD than in control cells. Indeed, Oil Red O staining showed increased accumulation of the neutral lipids triglyceride (TG) and cholesteryl esters (CE; Fig. 2A). Further analysis by lipidomics confirmed that intracellular TG concentration increased ∼4-fold, from 7.2 ± 1.1 pmol/μg protein in control cells to 29 ± 7 pmol/μg of protein in *LRP1* KD cells (Fig. 2B, *P*< 0.001). CE concentration increased by almost 3-fold, from 56 ± 11 pmol/μg protein in control cells to 146 ± 52 pmol/μg protein in *LRP1* KD cells (*P*< 0.005, Fig. 2B, mean ± SD).

The TG increase in *LRP1* KD cells was associated with reduced mRNA expression of the upstream regulator of FA oxidation *PPARa* by ∼50% (*P*< 0.05, Fig. 2C), as well as of mitochondrial carnitine palmitoyltransferase I (*CPT1*) by ∼95%, and of acyl-CoA oxidase (*ACOX1*) by 55% (*P*< 0.01 for both, Fig. 2C), suggesting decreased capacity for lipid oxidation. Consistently, acylcarnitine amounts were lower in *LRP1* KD cells than in control cells (Fig. 2D), despite the rate of fatty acid oxidation activity being preserved (Fig. 2E). The mRNA expression of pyruvate dehydrogenase kinase 4 (*PDK4*), which inhibits the entry of glucose-derived substrate into the mitochondria in favor of lipid-derived substrates, was increased in *LRP1* KD cells (Fig. 2F). Altogether, the gene expression and lipid data suggest multiple adaptations in mitochondrial activity in response to LRP1 loss, with decreased potential to use both glucose and lipid-derived substrates. There were no changes in mRNA expression of synthetic enzymes diacylglycerol acyltransferases (*DGAT1* and *DGAT2*), in the levels of TG precursors monoacylglycerol (MAG), PA and diacylglycerol (DAG) (Fig. 2G–H), or in uptake of FFA from the media between control and *LRP1* KD cells (Fig. 2I).

The intracellular accumulation of CE in *LRP1* KD was attributable to CE species CE 18:1, which increased from 6.8 ± 1.5 pmol/μg of protein to 19.2 ± 4.1 pmol/μg of protein (*P*< 0.001) and CE 22:6, which increased from 27 ± 5 pmol/μg of protein to 53 ± 21 pmol/μg of protein (*P*< 0.001; Fig. 2J, Supplemental Table S2, mean ± SD). This CE accumulation was not associated with increased intracellular free cholesterol (Supplemental Table S2) or with the mRNA expression of the synthetic enzymes HMG-CoA reductase and HMG-CoA synthase, which were unchanged. The mRNA expression of their upstream regulators *SREBP1* and *SREBP2*, which is modulated by their own expression and other transcriptional mechanisms (70), was decreased in *LRP1* KD cells (Fig. 2K). Detection by WB showed decreased protein levels of the cleaved, transcriptionally active, fragment of SREBP1 (Fig. 2L), consistent with the lower gene expression of its targets *FASN*, *ACC1* and *SCD1* (Fig. 1I). The cleaved fragment of SREBP2 did not show differences between control and LRP1 KD cells, despite the full-size protein levels being lower in LRP1 KD cells (Fig. 2M). These data were consistent with the mRNA levels of SREBP2 targets HMG-CoA reductase and HMG-CoA synthase (Fig. 2K) and suggested that cholesterol synthesis was not impacted by LRP1 levels.

Cell culture media clearance of Dil-labeled LDL, a source of extracellular cholesterol, was not different between control and *LRP1* KD cells (Fig. 2N). Conversely, the mRNA expression of cholesterol exporters *ABCA1* and *ABCG1* decreased by ∼50% in *LRP1* KD cells (*P*< 0.05 and *P*< 0.01, respectively, Fig. 2O). The concentration of CE remaining in the media after an overnight incubation with the cells was significantly lower for LRP1 KD: 97 ± 18 pmol/μg protein in controls versus 41 ± 8 pmol/μg protein in *LRP1* KD (*P*< 0.001; mean ± SD; Fig. 2P). These data suggest decreased cholesterol turnover in *LRP1* KD cells, resulting in intracellular CE accumulation and more avid depletion of cholesterol from the *LRP1* KD cell culture media than in control cells.

Together, the data show that loss of LRP1 decreased expression of cholesterol exporters, increased storage of intracellular TG and CE, and lowered SREBP1 activation.

### Loss of LRP1 in T2C impaired surfactant lipid metabolism in female mice

**Fig. 3:**
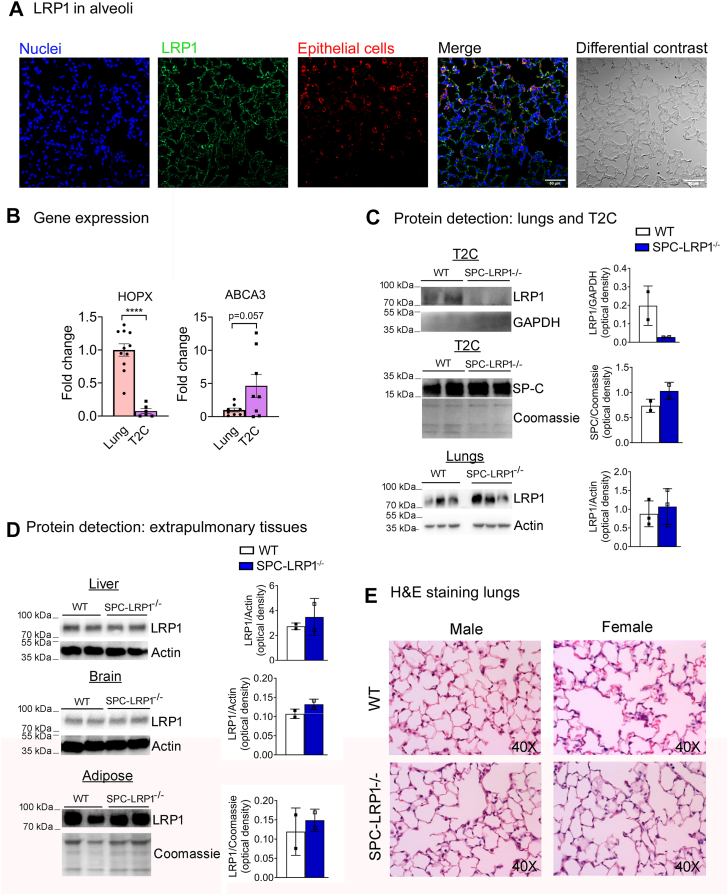
Generation of T2C-specific LRP1 knockout (SPC-LRP1−/−) mice. Mice floxed for LRP1 were crossed with mice expressing tamoxifen-inducible Cre recombinase under the control of SP-C promoter. Recombination was induced at 5–7 weeks of age and tissues were collected after a minimum of 4 weeks. Floxed mice without Cre expression were used as WT. A: Immunofluorescence for detection of LRP1 in alveoli. Cell nuclei were labelled with Hoechst (blue), LRP1 with Alexa-488-conjugated antibodies (green), and epithelial cells with antibodies against EpCam and Alexa-555-conjugated (red). Overlap and differential interference contrast images are shown too. B: Gene expression of bronchoalveolar epithelial and alveolar type 1 marker HOPX and T2C marker ABCA3 in lung homogenates and in primary T2C-enriched fractions isolated from mice. C: Western blot detection of LRP1 in isolated T2C and lung homogenates from WT and SPC-LRP1^−/−^ mice. D: Western blot detection of LRP1 in homogenates of liver, brain and adipose harvested from WT and SPC-LRP1^−/−^ mice. For all blots, equal amounts of protein were loaded in each lane and detection for the housekeeping proteins actin or GAPDH and Coomassie staining were used as loading controls. Optical density quantification is shown next to each blot. E: Representative images of H&E staining showing parenchymal areas from lungs of WT and SPC-LRP1^−/−^ mice.

We next sought to investigate the role of T2C LRP1 in vivo. LRP1 is widely expressed in lung parenchyma, as visualized by immunofluorescence in mouse lungs, and it is detectable in alveolar epithelial cells (Fig. 3A). We generated T2C-specific *Lrp1* knockout mice (SPC-LRP1^−/−^) by crossing *Lrp1*^*flox/flox*^ mice with mice expressing tamoxifen-inducible Cre recombinase under the control of the promoter for SP-C, expressed specifically in T2C. Tamoxifen was injected after completion of post-natal lung development. T2C were isolated and the purity of the isolated fraction was confirmed by qPCR analysis for enrichment of T2C marker *Abca3* and minor detection of HOP homeobox (*Hopx*), a marker of T1C and other epithelial cells in the bronchoalveolar region (71), when compared with lung homogenates (Fig. 3B). The isolated T2C fraction was positive for SP-C and negative for the immune-cell marker CD45 assessed by WB. SPC-LRP1^−/−^ mice showed deletion of LRP1 protein only in isolated primary T2C (Fig. 3C), while maintaining unchanged LRP1 protein expression in other tissues, including lung (Fig. 3C), as well as liver, brain and adipose tissue (Fig. 3D). H&E staining of lung sections did not show any gross morphological differences between WT and SPC-LRP1^−/−^ mice (Fig. 3E).

**Fig. 4:**
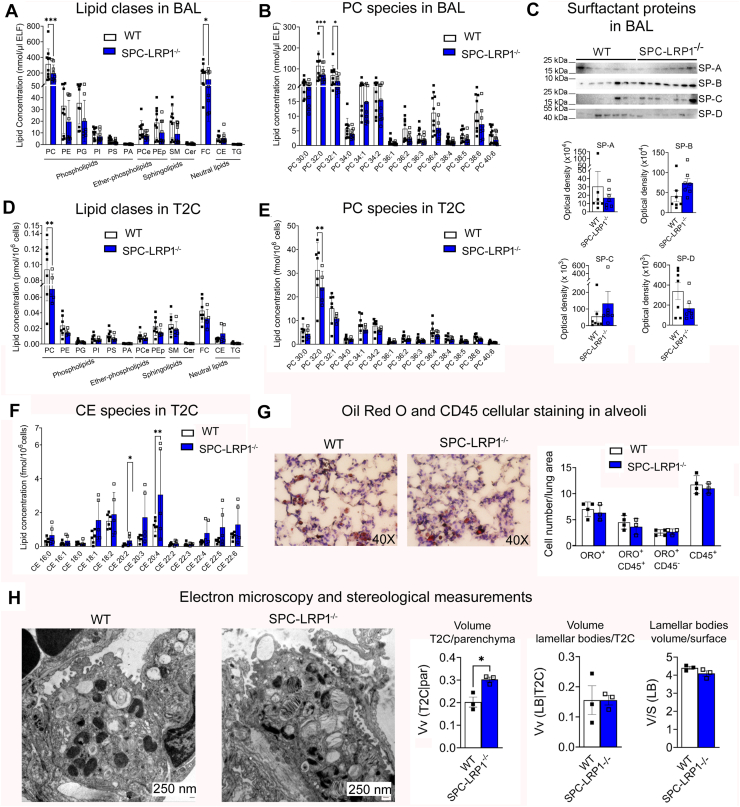
SPC-LRP1^−/−^ showed lower amounts of surfactant lipid than WT mice. T2C, BAL and lung tissue from WT and SPC-LRP1^−/−^ mice were analyzed for surfactant biology. A: Concentration of the different lipid groups and classes detected in BAL fluid. B: Concentration of the most abundant PC acyl-species detected in BAL fluid. C: Western blot for surfactant proteins in BAL fluid. Optical density quantification for each blot is shown below. D: Concentration of the different intracellular lipid groups and classes detected in primary T2C. E: Concentration of the most abundant PC acyl-species detected in T2C. F: Concentration of the most abundant CE acyl-species in T2C. G: Representative images of lung sections co-stained for neutral lipid by Oil Red O and for immune cells by CD45 immunohistochemistry. Count of cells that stained positive for each marker alone or both is shown. H: Representative transmission electron microscopy images of T2C. Stereological parameters were quantified and are shown in the left panels: volume of T2C relative to parenchyma (Vv (T2C|par)), volume of lamellar bodies relative to T2C (Vv(LB |T2C), and surface/volume ratio of lamellar bodies (S/V (LB)). Mean ± SD is shown. Significance: ∗*P*< 0.05, ∗∗*P*< 0.01, ∗∗∗*P*< 0.001. BAL, bronchoalveolar lavage: CD45, cluster of differentiation 45; CE, cholesteryl ester; Cer, ceramide; FC, free cholesterol; ORO, oil red O; PA, phosphatidic acid; PC, phosphatidylcholine; PCe, ether phosphatidylcholine; PE, phosphatidylethanolamine; PEp, plasmalogen phosphatidylethanolamine; PG, phosphatidylglycerol; PI, phosphatidylinositol; PS, phosphatidylserine; TG, triglyceride; SM, sphingomyelin; SP-A, surfactant protein A; SP-B, surfactant protein B; SP-C, surfactant protein C; SP-D, surfactant protein D.

Cell-free BAL was analyzed for surfactant lipid content. The surfactant composition in female but not in male mice showed reduced PL content, as we previously found in vitro. Supplemental Tables S3 and S4 show the amounts of all lipid classes and species identified in the BAL of female and male mice. BAL from female SPC-LRP1^−/−^ showed ∼30% decreased levels of PC: WT mice had 315 ± 193 pmol PC/μl ELF, versus 196 ± 107 pmol PC/μl ELF in SPC-LRP1^−/−^ mice (mean ± SD; *P*< 0.001, Fig. 4A and Supplemental Table S3). The decrease was attributable to lower amounts of the most abundant species, PC 32:0 and PC 32:1, but decreasing trends were observed for all PC species (Fig. 4B). In addition to PC, free cholesterol decreased from 194 ± 113 pmol/μl ELF in WT mice versus 119 ± 99 pmol/μl ELF (*P*< 0.001), and the main species of other lipid classes, such as phosphatidylglycerol (PG) 34:1 and 34:2, sphingomyelin (SM) 16:0 and 20:0, and ether-phosphatidylcholine (PCe) 32:0, 30:0 and 34:1 also showed significant decreases in BAL from SPC-LRP1^−/−^ mice (Supplemental Table S3). No differences were observed in lysophospholipids, suggesting that the decreased availability of PC was not due to extracellular degradation. Since surfactant proteins comprise ∼10% of the surfactant mass, we analyzed their abundance in BAL by WB. There were no differences in the BAL abundance of SP-A, SP-B, SP-C or SP-D between WT and SPC-LRP1^−/−^ mice (Fig. 4C). In contrast with female mice, male SPC-LRP1^−/−^ mice did not show a different BAL lipid composition than WT mice (Supplemental Table S4). Therefore, in female mice, loss of LRP1 in T2C decreased surfactant PL, while surfactant proteins were not affected.

**Supplemental Figure S2:**
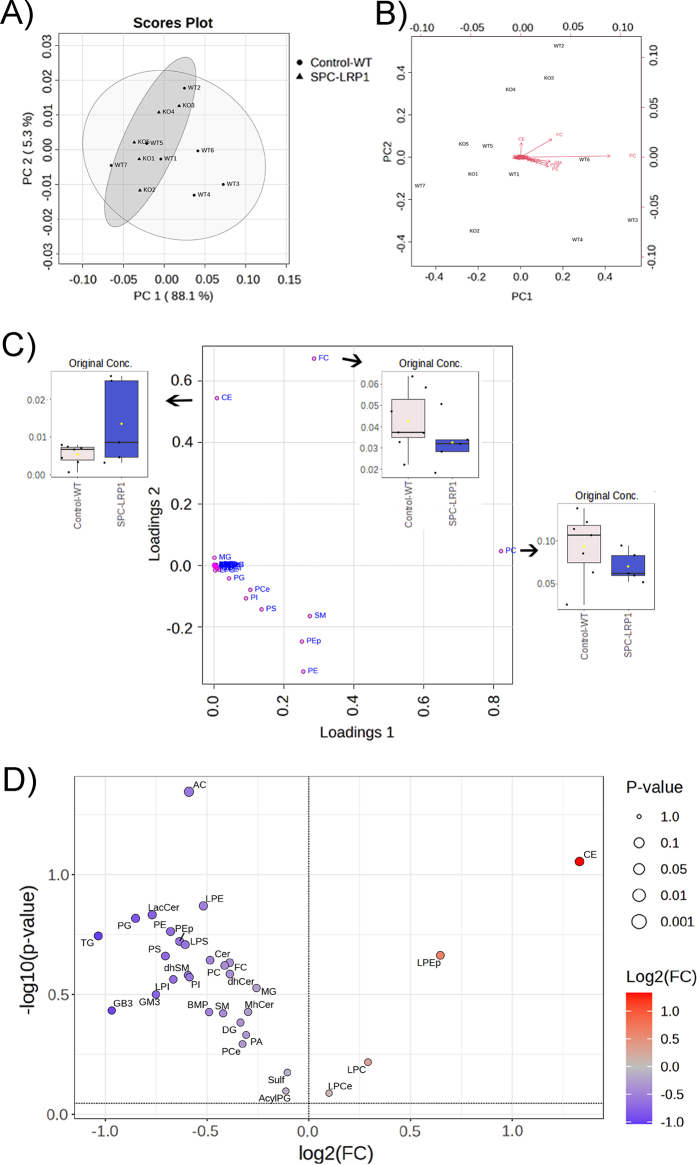


To determine whether the decreased availability of surfactant PC in BAL of female mice was associated with decreased intracellular lipid availability, lipidomic analysis of T2C isolated from the SPC-LRP1^−/−^ mice was compared to floxed controls. Unbiased principal component analysis (PCA) of the detected lipid classes showed separation of the WT and SPC-LRP1^−/−^ mice. Changes in PC, FC and CE were the most relevant features and accounted for more than 90% of the variance (Supplemental Fig. S2). Similar to the patterns encountered in vitro in *LRP1 KD* cells, primary T2C cells isolated from female SPC-LRP1^−/−^ mice showed lower intracellular PC content than those from WT mice: 0.094 ± 0.04 pmol/10^6^cells in WT mice versus 0.07 ± 0.02 pmol/10^6^cells in SPC-LRP1^−/−^ mice (*P*< 0.0001; Fig. 4D; Supplemental Table S5). This decrease was attributable to lower levels of the most abundant species, such as PC 32:0, which was 31.33 ± 11.5 fmol/10^6^cells in WT mice versus 23.97 ± 6.9 fmol/10^6^cells in SPC-LRP1^−/−^ mice (*P*< 0.001; Fig. 4E). All other PC species, PE, PCe, other PLs, free cholesterol and sphingomyelin all showed trends towards decreased abundance in T2C from SPC-LRP1^−/−^ mice (Fig. 4D; Supplemental Table S5). Therefore, the composition profile of intracellular T2C PLs mirrored that of extracellular BAL PLs in WT and SPC-LRP1^−/−^ mice.

Intracellular CE was the only major lipid group that showed a trend toward higher levels in T2C from SPC-LRP1^−/−^ mice (Fig. 4D; Supplemental Fig. S2D). When focusing on each CE species, CE 20:3 and CE 20:4 levels significantly increased by ∼3 fold in SPC-LRP1^−/−^ mice. All other CE species showed trends toward increased levels that did not reach statistical significance (Fig. 4F, Supplemental Table S5). Overall, the amounts of neutral lipid in T2C in vivo were lower than in our in vitro model, and therefore, differences were not detected in lung sections stained with Oil Red O (Fig. 4G).

Further characterization by transmission electron microscopy of lung tissue and stereological measurements showed that T2C in SPC-LRP1^−/−^ mice comprised a larger volume of the parenchyma than in WT mice, suggesting increases in cell number or size. Lamellar bodies did not show statistically significant differences in their volume per T2C or in their volume/surface ratio (Fig. 4H).

These data indicate that loss of LRP1 in T2C in vivo replicated in female mice the lipid phenotype previously observed in vitro, with decreased surfactant PL and increased intracellular CE.

### Female SPC-LRP1^−/−^ mice exhibit decreased pulmonary compliance

**Fig. 5:**
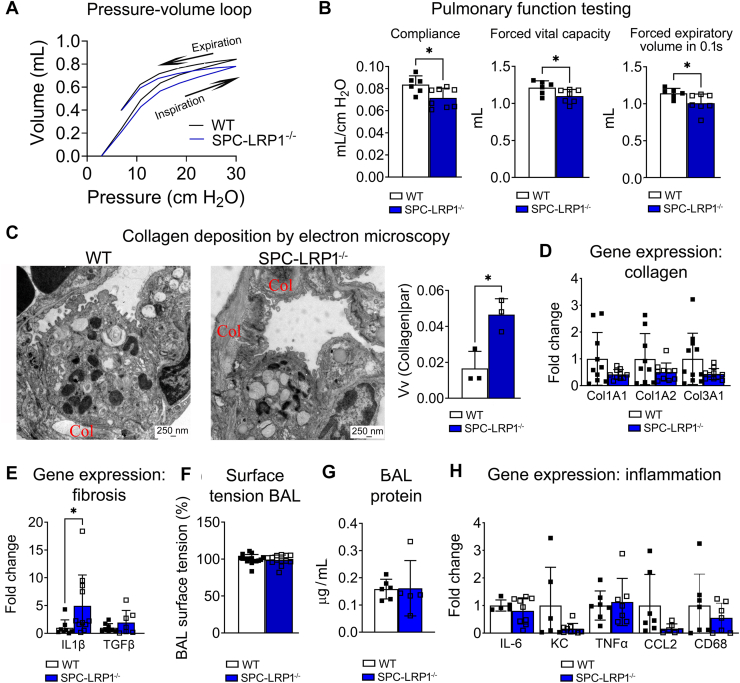
Female SPC-LRP1−/− mice showed less pulmonary compliance than WT mice. Pulmonary function, morphology and gene expression were analyzed in WT and SPC-LRP1^−/−^ mice. All data in this figure refer to female mice. A: Pressure-volume loops analyzed by forced oscillatory maneuvers (Flexivent, SciReq). Arrows indicate the direction of the loop for a given respiratory cycle. The bottom portion corresponds to the inspiratory phase, and the upper portion corresponds to the expiratory phase. B: Selected pulmonary function parameters: compliance, forced vital capacity (FVC) and forced expiratory volume in 0.1 s (FEV0.1). C: Representative transmission electron microscopy images highlighting collagen areas with the letters Col. Stereology-based quantification of the volume of collagen relative to parenchyma (Vv (Collagen|par)) is shown. D: Gene expression of the most common collagen components of the extracellular matrix, measured in lung homogenates. E: Gene expression of profibrotic markers in lung homogenates. F: Surface tension of BAL fluid, relative to the measurement in BAL from WT mice. G: Protein concentration in BAL fluid. H: Gene expression of proinflammatory markers in lung homogenates. Mean ± SD is shown. Significance: ∗*P*< 0.05. CCL2, chemokine ligand 2; CD68, cluster of differentiation 68; Col1A1, Collagen, type I, alpha 1; Col1A2, Collagen, type I, alpha 2; Col3A1, Collagen, type III, alpha 1; IL1β, interleukin 1 beta; IL-6, interleukin 6; KC, keratinocyte-derived cytokine; TGFβ, transforming growth factor beta; TNFα, tumor necrosis factor alpha.

To determine the physiological impact of decreased surfactant availability, pulmonary function testing was conducted. All respiratory parameter data are in Supplemental Table S6. Figure 5A shows the pulmonary pressure-to-volume relationship for WT and SPC-LRP1^−/−^ mice during the respiratory cycle while connected to a ventilator. In these curves, the lower branch of the loop corresponds to the inspiratory phase, during which alveolar air volume increases as the ventilator pressure rises to 30 cm H_2_O. The upper branch of the loop corresponds to the expiratory cycle, as the air in the lungs is expelled passively when the pressure is released. In SPC-LRP1^−/−^ female mice, the pressure-volume loop showed a shift towards the right and down (Fig. 5A). This is indicative of restrictive conditions, as SPC-LRP1^−/−^ mice require higher pressures to reach the same air volumes as WT mice. For every pressure point, the volume of intrapulmonary air was lower for SPC-LRP1^−/−^ mice than for WT mice. When measuring specific respiratory parameters, compliance (Cst) decreased from 0.08 ± 0.01 ml/cmH_2_O in WT mice to 0.07 ± 0.01 ml/cmH_2_O in SPC-LRP1^−/−^ mice and forced vital capacity (FVC) decreased from 1.21 ± 0.09 ml in WT mice to 1.10 ± 0.09 in SPC-LRP1^−/−^ mice (mean ± SD; *P*< 0.05 for both; Fig. 5B). These data indicate increased work of breathing and decreased air capacity in the lungs of SPC-LRP1^−/−^ mice than in those from WT mice. Forced expiratory volume in one second (FEV0.1) also decreased significantly in SPC-LRP1^−/−^ mice, but the ratio FEV0.1/FVC, a measure of COPD, stayed unchanged (Fig. 5B and Supplemental Table S6). In contrast with female mice, PFT measurements in male mice did not show significant differences between WT and SPC-LRP1^−/−^ (Supplemental Table S6).

We further analyzed SPC-LRP1^−/−^ female mice to assess whether the decreased lung compliance could be due to excessive deposition of extracellular matrix (ECM), loss of surfactant and associated increase in surface tension, or edema associated with inflammation. Electron microscopy revealed more collagen deposition in the alveolar subepithelial regions in SPC-LRP1^−/−^ mice (Fig. 5C). Indeed, the volume fraction of collagen in lung parenchyma increased from 0.016 ± 0.01 versus in WT mice to 0.046 ± 0.01 in SPC-LRP1^−/−^ mice (V|v Collagen/parenchyma; *P*< 0.05). Whole lung homogenate mRNA expression of collagens *Col1a1*, *Col1a2* and *Col3a1* did not differ between WT and SPC-LRP1^−/−^ mice (Fig. 5D), but SPC-LRP1^−/−^ mice had ∼5 fold higher expression of pro-fibrotic *Il1β* (*P*< 0.01; Fig. 5E). There were no differences between WT and SPC-LRP1^−/−^ mice in BAL surface tension, or in markers of inflammation, such as BAL protein concentration or mRNA expression of interleukin 6 (*IL-6*), chemokine ligand 1 (KC), tumor necrosis factor alpha (*Tnfa*), C-C motif ligand 2 (*Ccl2*) and the cluster of differentiation 68 (*Cd68*) (Fig. 5F–H). Together, these data indicate decreased compliance, with increased collagen deposition, and suggest a modest contribution of pro-inflammatory mediators to the phenotype of SPC-LRP1^−/−^ female mice.

### Cigarette smoke exposure decreased pulmonary function in male and female SPC-LRP1^−/−^mice

**Fig. 6:**
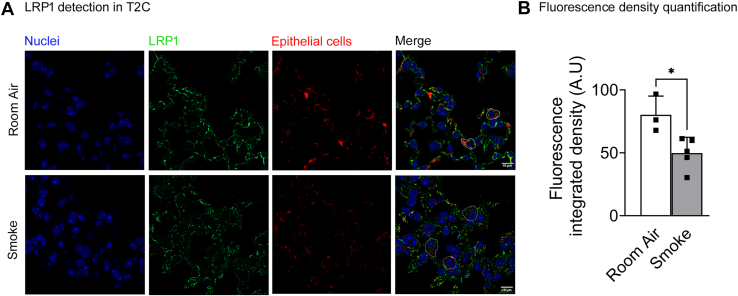
Detection of LRP1 in T2C in mice exposed to room air and smoke. Female WT mice were exposed to 6 months of room air or second-hand smoke, and lungs were collected, fixed, and processed for immunohistochemistry. A: Representative images obtained by confocal microscopy. Cell nuclei were labelled with Hoechst (blue), LRP1 with Alexa-488-conjugated antibodies (green), and epithelial cells with antibodies against EpCam and Alexa-555-conjugated (red). Images were acquired at 63X and alveolar epithelial cells with cuboidal morphology were selected as T2C as marked for some examples in the merged image. B: Green fluorescence, corresponding to LRP1, was quantified in the selected cells. Mean ± SD is shown. Significance: ∗*P*< 0.05.

**Fig. 7:**
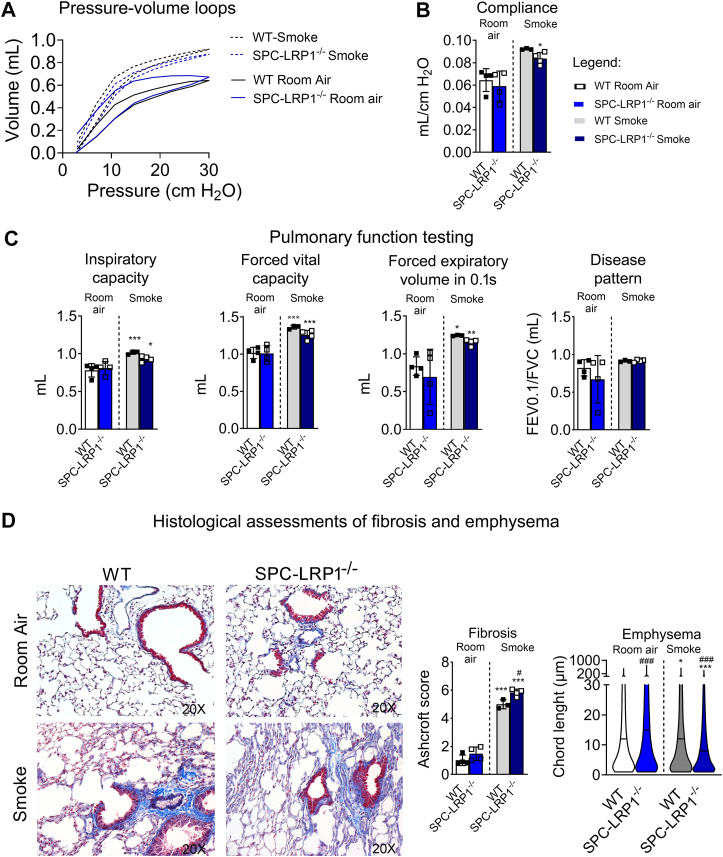
Female SPC-LRP1−/− mice had exacerbated smoke-induced fibrotic remodeling. WT and SPC-LRP1^−/−^ mice were exposed to second-hand smoke or room air for 6 months, starting at 2 months of age, and pulmonary function and morphology were evaluated. All data in this figure refer to female mice. A: Pressure-volume loops for WT and SPC-LRP1^−/−^mice exposed to smoke (dashed line) or room air (solid line). B: Pulmonary compliance for WT and SPC-LRP1^−/−^mice exposed to smoke or room air. C: Other relevant function testing parameters: inspiratory capacity, FVC, FEV0.1 and ratio FEV0.1/FVC for identification of disease pattern. D: Histological assessment of fibrosis and emphysema. Representative images of lung sections stained with Masson’s trichrome (left panels), fibrosis scoring using Ashcroft’s system, and chord length measurements. Mean ± SD is shown. Significance: ∗*P*< 0.05, ∗∗*P*< 0.01,∗∗∗*P*< 0.001 for comparisons between room air and smoke groups, within the same genotype, and ^#^*P*< 0.05, ###*P*< 0.001 for comparisons between WT and SPC-LRP1^−/−^, within the same exposure.

SNPs in *LRP1* were associated with decreased FEV1 and FEV1/FVC in smokers and in patients with COPD (14). We exposed WT mice to 6 months of room air or secondhand cigarette smoke, a mouse model of COPD, and used immunofluorescence to detect LRP1 in T2C. Quantification of fluorescence integrated density showed that mice exposed to smoke had less LRP1 in T2C than mice exposed to room air (Fig. 6). Since SNPs in LRP1 are often associated with decreased protein expression (9, 11, 12, 13, 14, 15, 16), we tested whether lower LRP1 expression in T2C could cause worse COPD outcomes. Female WT and SPC-LRP1^−/−^ mice were exposed to 6 months of room air or smoke, starting at 2 months of age, and pulmonary function was analyzed. As shown in Fig. 7, smoke exposure induced the expected obstructive pattern in mice, with a left-and-up shift for the P-V loops and increased compliance, IC, FVC and FEV0.1 in the smoke groups relative to the room air groups (Fig. 7A–C).

When compared to WT mice, SPC-LRP1^−/−^ female mice exposed to smoke maintained the trends previously observed at baseline, showing a shift to the right and bottom in the Pressure-Volume loop, indicative of a restriction (Fig. 7A). IC, Cst and FVC showed trends towards lower values in SPC-LRP1^−/−^ mice than in WT mice in both the room air and the smoke group (Fig. 7B–C and Supplemental Table S7). The ratio FEV/FVC, an indicator of tissue destruction in emphysema, did not differ between WT and SPC-LRP1^−/−^ mice or between room air and smoke conditions (Fig. 7C), suggesting the coexistence of mixed obstructive and restrictive patterns. Indeed, analysis of lung sections for emphysema by chord length measurement and for fibrosis by trichrome staining and Ashcroft scoring showed that female SPC-LRP1^−/−^ had more airspace enlargement than WT mice at baseline (*P*< 0.001), and worse fibrosis (*P*< 0.05) after the smoke challenge (Fig. 7F).

Only when exposed to smoke did male SPC-LRP1^−/−^ mice show a functional phenotype, with lower Cst than WT mice: 0.094 ± 0.006 ml/cm H_2_O in WT versus 0.085 ± 0.005 ml/cm H_2_O in SPC-LRP1^−/−^ (mean ± SD; *P*< 0.005; Supplemental Fig. S3). FEV0.05 and the ratio FEV0.1/FVC showed modest decreases without reaching statistical significance (Supplemental Table S8). The pressure-volume loops and the fibrosis Ashcroft scoring did not show differences when comparing SPC-LRP1^−/−^ with WT mice within the smoke group (Supplemental Fig. S3).

Therefore, in female mice, smoke exposure led to similar pulmonary mechanics outcomes in both WT and SPC-LRP1^−/−^ mice and worse tissue fibrotic remodeling in SPC-LRP1^−/−^ than in WT.

### SPC-LRP1^−/−^ transcriptomic analysis

**Fig. 8:**
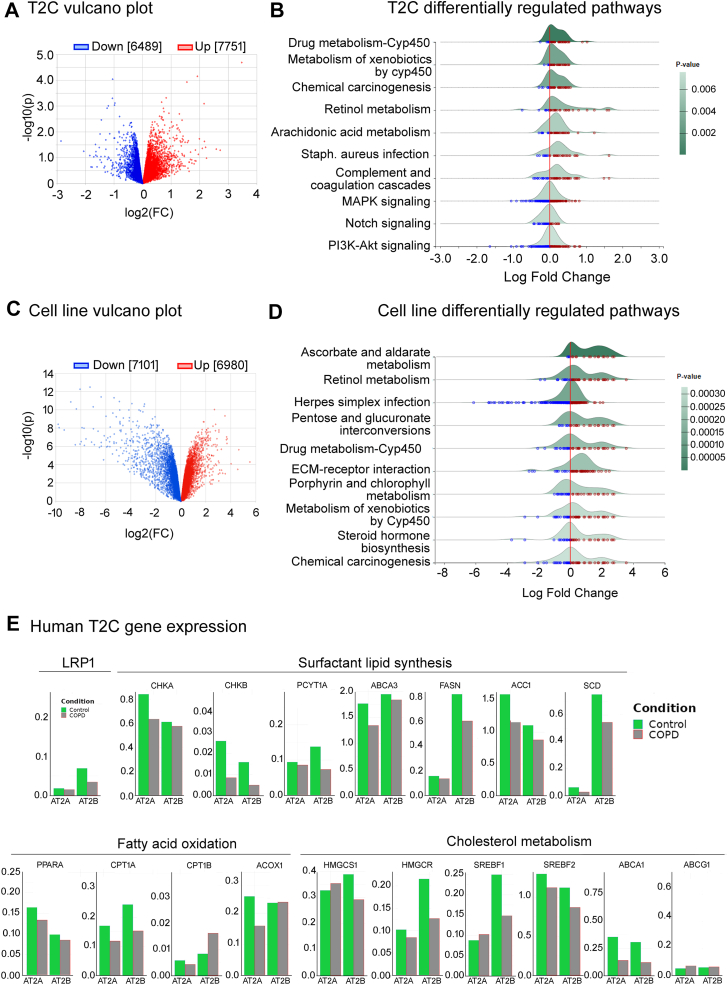
Transcriptomic analysis of T2C. Primary T2Cs were isolated from WT and SPC-LRP1^−/−^ mice and processed for RNA sequencing followed by pathway enrichment analysis. LRP1 KD and control cells were subjected to the same analysis. A: Vulcano plot showing all transcripts detected in mouse primary cells. Each dot represents a transcript. Red color indicates upregulation and blue color indicates downregulation in SPC-LRP1^−/−^ relative to WT mice. B: Ridgeline plot of KEGG pathways enriched in T2C from SPC-LRP1^−/−^ relative to WT mice analyzed by GSEA. Each dot represents a transcript in a specific pathway, and its log-fold change relative to WT mice is shown. Red color indicates upregulation and blue color indicates downregulation in SPC-LRP1^−/−^ mice. Peaks displaced to the right indicate upregulation of the overall pathway, and peaks displaced to the left indicate downregulation. Pathway regulation significance is indicated by darker shading of the peak’s area and the reference shading is shown on the right side. C: Vulcano plot showing all transcripts detected in LRP1 KD and control cell lines, using the same color-coding criteria as above. D: Ridgeline plot of KEGG pathways enriched in LRP1 KD cells, using the same color-coding criteria as above. E: Expression of LRP1 and enzymes involved in surfactant lipid synthesis, fatty acid oxidation and cholesterol metabolism in T2C populations of human samples from patients with COPD and control subjects in the publicly available dataset GSE136831. ABCA1, ATP Binding Cassette Subfamily A Member 1; ABCA3, ATP Binding Cassette Subfamily A Member 3; ABCG1, ATP Binding Cassette Subfamily G Member 1; ACC1, Acetyl-CoA carboxylase 1; ACOX1, acyl-Co oxidase 1; CHKA, choline kinase A; CHKA, choline kinase B; CPT1A, carnitine palmitoyltransferase 1 alpha; CPT1B, carnitine palmitoyltransferase 1 beta; FASN, fatty acid synthase; HMGCS1, hydroxymethylglutaryl-CoA synthase 1; HMGCR, hydroxymethylglutaryl-CoA reductase; PCYT1A, CTP:phosphocholine cytidylyltransferase alpha; PPARA, peroxisome proliferator-activated receptor alpha; SCD 1, stearoyl-CoA desaturase; SREBF, sterol regulatory element-binding factor.

To further determine the mechanism for LRP1 loss in T2C leading to compromised pulmonary function in 6-month-old female mice, we conducted unbiased transcriptomic analysis of T2C isolated from female mice at an early time point, at 12 weeks of age. Figure 8A shows a volcano plot of all the detected transcripts. A total of 14,240 transcripts were mapped, of which 45% were downregulated and 55% upregulated in T2C from SPC-LRP1^−/−^ mice when compared with T2C from WT mice. Pathway enrichment analysis was conducted using the GSEA method and the KEGG database to group the transcripts into pathways (72). The top 10 most significantly enriched pathways are shown in Fig. 8B, and they could be grouped into major biological categories as follows: 1) Pathways related to biodegradation and metabolism of xenobiotics: “Drug metabolism-cytochrome P450”, “Metabolism of xenobiotics by cytochrome P450”, and “Chemical carcinogenesis”; 2) Lipid-related pathways: “Retinol metabolism” and “Arachidonic acid”; 3) Infection and immunity-related pathways “Staphylococcus aureus infection” and “Complement and coagulation cascades”. In each of these pathways, most of the detected transcripts were up to 10-fold higher in T2C isolated from SPC-LRP1^−/−^ mice than in T2C from WT mice (Fig. 8B). Thus, the pathways were upregulated, with positive enrichment scores (ES) ranging from 0.56 to 0.69, and *P* values ranging from 0.001 to 4.5·10^-5^. The signal transduction pathways “MAPK signaling pathway”, “Notch signaling pathway” and “PI3K-Akt signaling pathway” contained both overexpressed and underexpressed transcripts, with ES ranging from −0.28 to −0.46 and *P* values between 0.004 and 0.006, indicating modest downregulation (Fig. 8B). These data suggest that, at baseline, the most significant transcriptional changes caused by loss of LRP1 were upregulation of detoxification, lipid signaling and inflammatory pathways.

We focused on the physiological meaning of each pathway. Table 4 summarizes the pathway analysis, showing representative transcripts for each pathway, as well as relevant information on their substrates, products and potential downstream effects. Xenobiotic and drug metabolism pathways, the most significantly upregulated, use substrates such as nicotine and aromatic compounds of natural or artificial origin. The products are phase I and phase II metabolites conjugated to glutathione or glucuronic acid. The pathway of arachidonic acid metabolism included transcripts from the family of phospholipase A2 (*Pla2*) and of eicosanoid synthesis by arachidonic acid lipooxygenase (*Alox*), cyclooxygenase (*Ptgs*/*Cox*), and *Cyp*. Notably, *Cox2* was downregulated, while transcripts in the other two branches of the pathway were upregulated. Upregulation of the retinol metabolism pathway was due to enrichment in transcripts encoding enzymes involved in the metabolism of all-*trans*-retinoic acid (ATRA). These included retinol dehydrogenases (*Rdh10*, *Rdh1*) that catalyze the oxidation of retinol to retinaldehyde, retinaldehyde dehydrogenases (*Aldh1a1*, *Aldh1a2*) that catalyze the synthesis of ATRA from retinaldehyde, and cytochrome P450 enzymes involved in specific ATRA catabolic oxidation (such as *Cyp26b1*). Other upregulated *Cyp* enzymes can also contribute to ATRA oxidation in non-specific manners (such as *Cyp1a1*, *Cyp2b10*, *Cyp3a13*, *Cyp4a32*, and *Cyp2s1*) (73). The pathways “Staphylococcus aureus infection” and “Complement and coagulation cascades” constitute innate immune responses and share multiple transcripts, including complement factors and adhesion and chemotactic molecules. The downregulated transcripts in the Notch signaling pathway included Notch ligand isoforms 1–4, canonical ligand Delta and Jagged, and *Adam17*. “PI3K-Akt signaling” and “MAPK signaling” pathways had an overall negative ES, but they integrate multiple potential cellular responses and the direction of change for each possible branch was unclear. Overall, the transcriptomic analysis of T2C from SPC-LRP1^−/−^ mice revealed activation of detoxification and inflammatory pathways and modest downregulation of the Notch signaling pathway.

**Table 4:** Description of significantly regulated pathways in T2C isolated from SPC-LRP1^−/−^ mice

Pathway	Representative Transcripts	Substrates	Products	Downstream Effect
Xenobiotics degradation and metabolism: Drug metabolism-cytochrome P450. Chemical carcinogenesis. Metabolism of xenobiotics by cytochrome P450.	Cytochrome P450 (*Cyp*), Glutathione-S-transferase (*Gst*), flavin containing monooxygenase (*Fmo*), aldehyde oxidase (*Aox*), UDP glucuronosyltransferase (*Ugt*), monoamine oxidase (*Mao*), and carbonyl reductase (*Cbr*).	Drugs, toxins, carcinogens such as nicotine, aflatoxin and aromatic compounds.	Phase I and phase II metabolites conjugated to glutathione or glucuronic acid.	DNA adducts. Carcinogenesis.
Infection and immunity: Staphylococcus aureus infection. Complement and coagulation cascades.	Complement factors, adhesion and chemotactic molecules.	Virus, bacterial components, coagulation factors.	Signaling cascades.	Innate immunity. Activation of immune cells.
Retinol metabolism	Retinol dehydrogenase (*Rdh*), short chain dehydrogenase/reductase (*Dhrs*), retinal dehydrogenase (*Aldh*), lecithin-retinol acyltransferase (*Lrat*).	Retinol (vitamin A).	Retinol ester, all-trans-retinoic acid, degradation products.	Gene transcription.
Arachidonic acid metabolism	Phospholipase A2 (*Pla2*), arachidonic acid lipooxygenase (*Alox*), cyclooxygenase (*Ptgs*/*Cox*), *Cyp*, epoxide hydrolase (*Epx*), and glutathione peroxidase (*Gpx*).	Phospholipid-derived arachidonic acid.	Bioactive eicosanoid lipids prostanoids, leukotrienes and epoxyeicosanoids.	Inflammation. Chemotaxis.
Notch signaling	Notch ligand isoforms 1–4, canonical ligand Delta and Jagged, and ADAM17.	Notch ligand.	Notch intracellular domain (transcription).	Cell communication, homeostasis and differentiation.
PI3K-Akt signaling MAPK signaling	Mitogen activated kinases (*Map3k11*, M*apk11*, *Mapk3*), growth factors (*Tgfb1*, *Vegfd*), phosphoinositide 3-kinase (*Pik3r6*), protein phosphatases (*Ppp2r5c*, *Ppp2r2c*).	Extracellular and intracellular ligands.	Signaling cascades.	Integration of cellular responses. Anabolism.

We then confirmed the consistency of our models by conducting the same analysis in scrambled and *LRP1* KD cells cultured at the ALI. The volcano plot showed ∼50% of transcripts upregulated and downregulated with *LRP1* KD (Fig. 8C). The differentially upregulated pathways were similar to those in mouse primary T2C, and had ES ranging from 0.53 to 0.88, and *P* values below 0.003 in all cases (Fig. 8D). Amongst the top 10 regulated pathways, there was upregulation of the same xenobiotic-related pathways as in mice, in addition to other related ones like “Ascorbate and aldarate metabolism”, and “Pentose and glucuronate interconversions”, which result in synthesis and degradation of glucuronic acid and its conjugates (72, 74), as well as “Porphyrin and chlorophyl metabolism”, which in human cells results in synthesis and metabolism of the heme group present in the cytochromes that participate in the pathways above (75). The “Retinol metabolism” pathway was upregulated in *LRP1* KD cells, consistent with T2C in SPC-LRP1^−/−^ mice. The only pathways specific to the human in vitro model were “Steroid hormone biosynthesis” (upregulated), where the upregulated transcripts were involved in the inactivation and conjugation of steroid compounds, “ECM-receptor interaction” (upregulated), and “Herpes simplex infection” (downregulated), suggesting the involvement of immune responses as in mice. This analysis revealed that our in vitro model of LRP1 KD cell cultures consistently replicates the intracellular transcriptional response to LRP1 loss observed in vivo in mouse T2C. The direction of change and the subcellular functions of the pathways were highly similar for mouse primary T2C and human cell lines.

The transcriptomic analysis above suggested mechanisms underlying the association between LRP1 expression and risk of developing COPD: most of the pathways impacted by LRP1 loss in our in vivo and in vitro models are known to be involved in COPD in humans (reviewed in (76, 77)). To confirm whether the lipid metabolic genes also changed in T2C from humans with COPD, we interrogated the publicly available scRNAseq data at copdcellatlas.com (NCBI Gene Expression Omnibus accession code GSE136831) (78). This human dataset identified two subpopulations of T2C: subpopulation A was characterized by their proliferative and regenerative potential, and subpopulation B was characterized by their high expression of surfactant proteins and mitochondrial and oxidative stress-related genes (78). Our interrogation of the database showed that LRP1 expression was lower in subpopulation B of T2C from patients with COPD than in control samples (Fig. 8E). Subpopulation B of human COPD patient-isolated T2C also showed lower expression of genes involved in surfactant lipid synthesis, such as *CK*, *CCTa*, *ABCA3*, *FASN*, *ACC1* and *SCD1* than in control subjects, similar to our in vitro studies with human cells comparing control cells (scrambled shRNA) and LRP1 KD cells. Further database interrogation of subpopulation B for genes involved in fatty acid oxidation, such as *PPARa* and *CPT1A*, and for genes involved in cholesterol metabolism, such as *HMGCS1*, *HMGCR*, *SREBF1*, *SREBF2* and *ABCA1*, showed lower expression in T2C from patients with COPD than in controls. While in this database, subpopulation B showed the most striking differences between patients with COPD and controls, subpopulation A of T2C followed the same trends (Fig. 8C). These patterns of gene expression were consistent with those we observed in *LRP1* KD cells (Figs. 1 and 2), highlighting the translational relevance of this model.

Together, the transcriptomic data show that T2C from COPD patients have lower expression of *LRP1* and other lipid metabolic genes than T2C from control subjects, and that loss of LRP1 in T2C results in overexpression of detoxification and inflammatory pathways in vitro and in vivo in mice. This analysis links LRP1 expression in T2C with surfactant lipid metabolism and COPD pathogenesis.

## Discussion

**Fig. 9:**
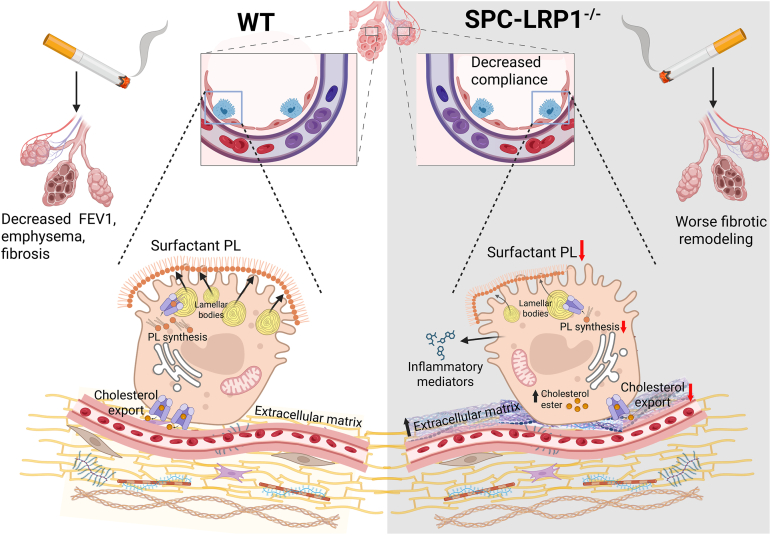
Summary of the findings in this study. Left side: in WT mice, T2Cs maintain tightly regulated metabolism, including surfactant PL synthesis and secretion, cholesterol export and maintenance of extracellular matrix, to ensure optimal surfactant and pulmonary function. Smoke exposure triggers alveolar tissue emphysematous destruction, increased ECM deposition and impairments in pulmonary function. Right side: in mice with decreased LRP1 expression in T2C (SPC-LRP1^−/−^ mice), lipid metabolism is suppressed, surfactant synthesis is compromised, and metabolic pathways involved in inflammation and xenobiotic detoxification are activated. With smoke exposure, such baseline results in exacerbated collagen deposition, and pulmonary fibrotic remodeling. Created in BioRender. Garcia, I. (2026) https://BioRender.com/xya5fb0.

*LRP1* genetic polymorphism variants are associated with multiple human diseases, but the molecular mechanisms for such associations are not understood (14, 79, 80). In smokers and COPD patients, the variant rs11172113 in *LRP1* is associated with decreased pulmonary function (14). This variant is also associated with coronary artery disease, migraine, and cervical artery dissection. In the aorta, adipose and smooth muscle cells, this DNA locus binds to transcription factors that regulate LRP1 protein expression, and it has been proposed to modulate genetic risk for vascular diseases (8, 16). Other variants that result in decreased LRP1 expression are also associated with negative effects on liver cholesterol homeostasis (13). T2C lipid metabolism undergoes smoke-induced reprogramming through multiple mechanisms (81), and patients with COPD have lower levels of surfactant lipids (33, 37). We hypothesized that the association of *LRP1* genetic variants with COPD may rely on the role of LRP1 in lipid metabolism and asked whether loss of LRP1 in alveolar T2C would alter intracellular lipids and surfactant metabolism, and negatively affect pulmonary function. Using our generated models of LRP1 loss, we found that 1) LRP1 is needed for surfactant phospholipid availability in vitro and in vivo in female but not in male mice, 2) smoke exposure decreased LRP1 protein in T2C in mice, 3) loss of LRP1 in T2C caused a restrictive pulmonary function defect at baseline in female mice, and it exacerbated smoke-induced tissue remodeling, 4) LRP1 loss in T2C upregulated subcellular detoxification and inflammatory pathways, and 5) T2C from humans with COPD have lower mRNA expression of LRP1 and of other lipid metabolic genes than T2C from control subjects. Figure 9 depicts a diagram with the main conclusions of the study.

*LRP1* KD cells showed suppression of lipid metabolism and surfactant PL consistent with observations in mice with loss of function of CCTα or ABCA3, which also show surfactant deficiencies and compromised pulmonary function (44, 47). *LRP1* KD cells also exhibited lower expression of ABCA1 and ABCG1 than control cells, as well as intracellular CE accumulation. Consistently, T2C from SPC-LRP1^−/−^ mice also accumulated CE. Lungs have a high expression of the PL and cholesterol exporters ABCA1 and ABCG1, and their deficiency alters surfactant lipids and pulmonary function in mice (45, 46), indicating their involvement in T2C lipid metabolism. ABCA1 and ABCG1 export cholesterol to circulating ApoA1 and HDL. In hepatocytes, loss of *Lrp1* impaired cholesterol export by decreasing the localization of ABCA1 on the plasma membrane in mice (20), and it caused low plasma HDL levels in humans (13). In vascular smooth muscle cells, loss of LRP1 increased intracellular CE levels and decreased ABCA1 expression in mice (82). Overall, specific loss of LRP1 in cells with high lipid metabolism activity disrupts intracellular homeostasis and results in a systemic phenotype consistent with the cellular function (79). Some of those phenotypes are consistent with our observations in T2C, which require active lipid metabolism to accomplish pulmonary surfactant production. Other T2C cellular functions involving lipids, such as mitochondrial fatty acid oxidation and PL transport by ABCA3 from the ER into lamellar bodies, also undergo strict regulation that is often disrupted during the pathophysiology of multiple pulmonary diseases (83, 84). Our study did not directly focus on lamellar body or mitochondrial biology, but the downregulation of *PPARa* and *CPT1b*, together with the lower levels of acyl-carnitines in *LRP1* KD cells could point to compromised mitochondrial function during conditions of cellular stress.

Pulmonary function in SPC-LRP1^−/−^ mice showed a mild restrictive phenotype with measurable loss of compliance, but collagen deposition was detectable only at electron microscopy magnification, and there were no other signs of disease at baseline. After a smoke exposure challenge, a model for studying COPD in mice, SPC-LRP1^−/−^ female mice showed worse fibrotic remodeling than WT mice, suggesting a link between LRP1 expression in T2C and pulmonary tissue remodeling. In mice, smoke exposure can trigger airway remodeling, emphysema or both (85). In humans, smoking can cause emphysema as well as combined pulmonary fibrosis and emphysema (CPFE) (86), and mutations in surfactant protein C and *ABCA3* genes are associated with a higher incidence of CPFE (87, 88). There was sexual dimorphism in the knockout mice phenotype, and at baseline, only female mice showed lower surfactant lipids and decreased compliance. Smoke exposure triggered fibrotic remodeling combined with emphysematous tissue destruction in female mice and decreased compliance in male mice. These data agree with the epidemiological data on COPD showing a higher prevalence in female patients, although the reasons for the different patterns are unclear (89).

The unbiased computational analysis of the transcriptome in primary T2C isolated from female mice aimed at elucidating the intracellular impact of LRP1 loss at an early time point, seeking a mechanism for the pulmonary function defects at later time points and the exacerbated remodeling after the smoke challenge. T2C from SPC-LRP1^−/−^ mice and human LRP1 KD cells displayed baseline overexpression of pathways that were previously shown to be associated with COPD and fibrosis. Oxidative stress and xenobiotic metabolism, which also increased in mice with airway epithelium-specific LRP1 loss (27), and metabolism of arachidonic acid, which produces proinflammatory and chemotactic lipids, are part of the pathogenic process in COPD (76, 90, 91). Consistent with our RNAseq data, increased activity of PLA2 and arachidonic acid levels were previously observed in LRP1-deficient smooth muscle cells (82). These detoxification pathways metabolize endogenously produced chemoattractant and inflammatory bioactive lipids, as well as exogenous xenobiotics like nicotine and tobacco-specific compounds (77). In some instances, the resulting products can react with cellular components and trigger known mechanisms of cellular dysfunction, such as the oxidation of proteins and lipids in different organelles and the generation of DNA adducts in the nuclei (92). The overall analysis suggests that LRP1 loss raises the baseline expression of these pathways, increasing cellular susceptibility to smoke-induced damage and ultimately worsening physiological outcomes in SPC-LRP1^−/−^ mice after the smoke challenge. The upregulation of the retinol metabolism pathway suggests activation of processes aimed at regenerating and restoring the altered function of T2C lacking LRP1. Retinol metabolism is centered around maintaining the cellular homeostasis of all-*trans*-retinoic acid (ATRA), which is a transcriptionally active retinoid that enables T2C proliferation, differentiation and surfactant production (93, 94, 95, 96, 97, 98, 99, 100). Studies using animal models have provided experimental evidence for the role of ATRA in lung regeneration and alveolarization through mechanisms that are still unclear (96, 101, 102, 103, 104, 105, 106, 107, 108, 109, 110, 111, 112).

Finally, we describe a simple model for in vitro study of surfactant lipid metabolism. Studying T2C in vitro is technically challenging because cellular polarity is lost in submerged cultures. Some studies have shown that A549 cells acquire ABCA3-expressing lamellar bodies, express SP-C and secrete phospholipid when cultured on a collagen matrix (65, 67, 68, 69). We added the ALI component for further cellular polarization and collection of lipids secreted to the apical side without mixture with the lipids of the culture media. *LRP1* KD cells showed more dramatic lipid changes than primary T2C from SPC-LRP1^−/−^ mice, and this discrepancy could be attributed to a higher degree of specificity in vivo than in vitro, and the inherent differences in cell microenvironments between the two systems (113). The main effects of LRP1 loss on T2C surfactant lipid availability were nonetheless consistent in both models, thus validating the in vitro system.

In summary, genetic variants in LRP1 are associated with a greater risk of COPD, and patients with COPD show lower expression of *LRP1* in surfactant-producing T2C than controls. Our data show that reduced LRP1 expression is pathological. In mice, T2C-specific loss of LRP1 impaired surfactant lipid availability, increased cellular expression of detoxification and inflammatory pathways, and worsened tissue remodeling after a smoke challenge. While loss of LRP1 expression had a modest impact on baseline pulmonary function, the interaction of this genetic trait with the environmental insult of smoking resulted in worse disease outcomes. We conclude that LRP1 expression in alveolar T2C links surfactant lipid metabolism, pulmonary function, and tissue remodeling after smoke exposure, revealing an axis that explains the genetic association of *LRP1* with worse COPD.

## Data availability

Raw data from the RNA sequencing have been deposited in the Gene Expression Omnibus (GEO) repository, with accession number GSE297307 for scrambled and LRP1 KD cell line data, and GSE296981 for Control and SPC-LRP1^−/−^ mice. All other data are included in the manuscript and Supplemental tables.

## Supplemental data

This article contains supplemental data.

## Conflict of interest

The authors declare that they do not have any conflicts of interest with the content of this article.

## Figures and Tables

**Supplemental Figure S3:**